# Enantiosensitive steering of free-induction decay

**DOI:** 10.1126/sciadv.abq1962

**Published:** 2022-06-15

**Authors:** Margarita Khokhlova, Emilio Pisanty, Serguei Patchkovskii, Olga Smirnova, Misha Ivanov

**Affiliations:** 1 Max Born Institute, 12489 Berlin, Germany.; 2 Department of Physics, King’s College London, WC2R 2LS London, UK.; 3 Technische Universität Berlin, 10623 Berlin, Germany.; 4 Department of Physics, Humboldt University, 12489 Berlin, Germany.; 5 Blackett Laboratory, Imperial College London, SW7 2AZ London, UK.

## Abstract

Chiral discrimination, a problem of vital importance, has recently become an emerging frontier in ultrafast physics, with remarkable progress achieved in multiphoton and strong-field regimes. Rydberg excitations, unavoidable in the strong-field regime and intentional for few-photon processes, arise in all these approaches. Here, we show how to harness this ubiquitous feature by introducing a new phenomenon, enantiosensitive free-induction decay, steered by a tricolor chiral field at a gentle intensity, structured in space and time. We demonstrate theoretically that an excited chiral molecule accumulates an enantiosensitive phase due to perturbative interactions with the tricolor chiral field, resulting in a spatial phase gradient steering the free-induction decay in opposite directions for opposite enantiomers. Our work introduces a general, extremely sensitive, all-optical enantiosensitive detection technique that avoids strong fields and takes full advantage of recent advances in structuring light.

## Teaser

Detection of molecular chirality using only the electric field of light becomes possible via a nonlinear Stark shift.

## INTRODUCTION

Chiral recognition is an essential task in chemistry (*1*), whose origin dates back to the birth of the discipline (*2*) with the discovery of the optical activity of biomolecules: In solution, different enantiomers, which are nonsuperimposable mirror images of each other, can rotate in opposite directions the polarization of light that travels through the medium. However, for dilute media and in gas phase, this linear optical effect is severely challenging to implement, because it relies on rather weak optical magnetic interactions that scale as the ratio of the molecular size to the optical wavelength. This creates a strong demand for an optical chiral discrimination method that relies purely on dipole-interaction physics and is based only on the local electric field of the light, a goal that is not possible to achieve within linear optics (*3*).

A recent breakthrough in nonlinear optics has bypassed this barrier, showing that nonlinear optical processes can work as a key to molecular chirality using only electric field optical interactions (*4*–*7*). This revolution improves on previous work on chiral nonlinear optics (*8*–*14*) and has been accompanied by a wealth of other electric field methods coupled to other nonoptical observables (*15*–*22*). These approaches are generally constructed using the tools of strong-field and ultrafast physics, and they can therefore naturally take advantage of the rich toolbox of attosecond science (*23*–*25*) and nonlinear spectroscopy (*26*) and are able to access time scales and electronic excitation pathways that are not available to microwave spectroscopy methods (*12*). However, this advantage has come at the price of nonperturbative processes that require high intensities (*4*–*6*), and this, in turn, creates the need for delicate schemes that apply the recently found nonlinear chiral properties of the spatially structured electromagnetic field (*4*, *22*) without destroying or disturbing the molecule.

**Fig. 1.**
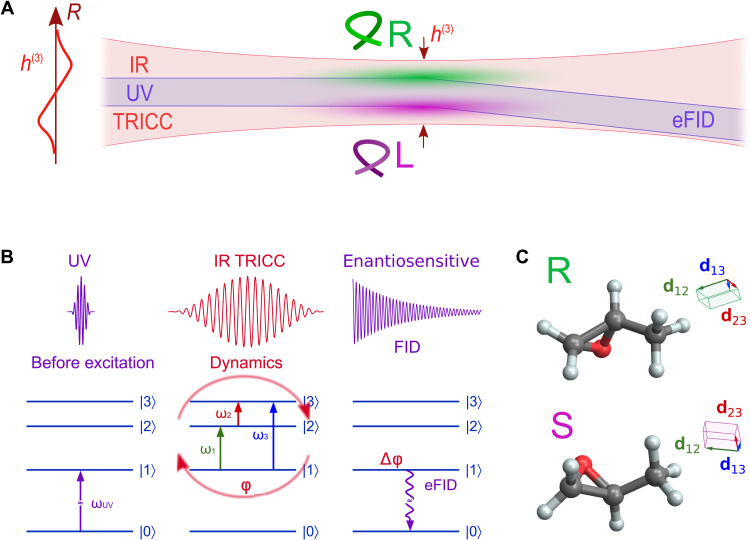
Enantiosensitive FID. (**A**) Focused ultraviolet (UV) and tricolor infrared (IR) beams interact with molecules in the focal region. Because of the focusing, this field becomes chiral, with the chiral correlation function *h*^(3)^ changing its sign across the focus (green and purple shades). The profile of *h*^(3)^ is shown on the left; the slope of *h*^(3)^ at the optical axis results in the redirection of the FID beam by chiral molecules. TRICC, tricolor chiral. (**B**) Scheme of the single-particle interaction. On the first step, the UV preexcites the molecule from the ground state ∣0⟩ into the excited state ∣1⟩. The TRICC field then induces dynamics between the excited states ∣1⟩, ∣2⟩, and ∣3⟩, resulting in an enantiosensitive quantum phase Δφ of the FID-active state ∣1⟩ after the TRICC pulse that steers the beam through the enantiosensitive FID (eFID). (**C**) R-methyloxirane and S-methyloxirane, and their three main transition dipoles, forming opposite chiral triplets.

In this work, we propose an experiment allowing chiral recognition on an ultrafast time scale using nondestructive weak fields. Our scheme builds on the recent advances in chiral synthetic light (*4*) to induce a controllable enantiosensitive quantum phase of the medium, which is then translated into easily measurable macroscopic observables by leveraging the exquisite control over light afforded by current progress in structured light (*27*) and in ultrafast control (*23*–*25*). Specifically, we adapt the ability to generate bright and coherent free-induction decay (FID) radiation (*28*–*30*) and, in particular, to steer it via quantum phase manipulation in atomic gases (*31*–*35*) to chirally sensitive drivers interacting with chiral media (see Fig. 1A), thereby introducing an enantiosensitive Stark shift that gives rise to FID labeling of enantiomers (FIDLE). Our approach is based on pure electric dipole physics and is therefore distinct from previous observations of chiral FID in dense media based on magnetic interactions (*36*).

At the microscopic level, we model a chiral molecule promoted from its ground state ∣0⟩ into an FID-active excited vibronic state ∣1⟩ by a coherent pump—in our case, a short ultraviolet (UV) pulse (see Fig. 1B)—and which reemits this photon energy by decaying back to the ground state. The phase of this emission, which is responsible for the direction of the resulting FID beam, is defined by the quantum phase of the FID-active state. The enantiosensitive contribution to the quantum phase is imparted by the enantiosensitive Stark shift arising in synthetic chiral fields. The simplest way to introduce the enantiosensitive Stark shift is to consider nonresonant interaction of the FID-active state ∣1⟩ with two other excited states, ∣2⟩ and ∣3⟩, induced by a tricolor chiral (TRICC) combination of infrared (IR) fields with frequencies ω_1_, ω_2_, and ω_3_ = ω_1_ + ω_2_ and noncolinear polarizations forming a chiral triplet. We create this chiral triplet macroscopically using tightly focused Gaussian beams to provide a longitudinal polarization component (*37*, *38*), resulting in a chiral time evolution of the electric field at every point. This construction corresponds to a nonlinear field chirality that changes sign across the focus, and this sign change is directly converted into the quantum phase of the FID-active state, thus steering the FID emission.

To demonstrate this, we first develop a general analytical theory of the chiral sensitivity of the TRICC FIDLE dynamics, and we suggest field configurations that produce the locally chiral electric fields required to drive these dynamics. We then present simulations of both near- and far-field observables for the methyloxirane molecule, showing a clearly visible enantiosensitive steering of the FID emission. Last, we explore the effect of the various TRICC-field parameters on this steering. Additional details and benchmarking are included in the Supplementary Materials.

## RESULTS

### Analytical theory

The core of our scheme is the chirally sensitive dynamics driven by the TRICC-field combination. We introduce these dynamics using a simple model of a molecule with three states involved, as shown in Fig. 1B. The system is driven by the TRICC pulse, consisting of three fields

$$E=\text{Re}[\sum_{j=1}^{3}E_{j}e^{-i(\omega_{j}t+\phi_{j})}]$$ (1)

(described in detail below) with a slowly varying envelope, which induces transitions between each pair of states ∣1⟩, ∣2⟩, and ∣3⟩. The time-dependent Schrödinger equation (TDSE) is solved fully analytically in both the resonant and off-resonant cases (see Methods and the Supplementary Materials). In both cases, the complex amplitude of the FID-active state is presented in the form *c*_1_(*t*) = *e*^*i*δ*Et*^, in the interaction picture with respect to the molecular Hamiltonian, where δ*E* is an energy shift resulting in the phase $\Delta \varphi =\int_{0}^{2\tau }\delta \mathrm{Edt}$ accumulated during the TRICC pulse with duration 2τ [and, given the sine-squared envelope, with full width at half maximum (FWHM) τ].

In the off-resonant case, the energy shift is written as

$$\delta E=2\frac{|V_{12}\|V_{13}\|V_{23}|}{\omega_{12}\omega_{13}}\;\text{cos}\;\phi +\frac{{|V_{12}|}^{2}}{\omega_{12}}+\frac{{|V_{13}|}^{2}}{\omega_{13}}$$ (2)

where $V_{12}=d_{12}\cdot E_{1}e^{i\phi_{1}}/2$, $V_{23}=d_{23}\cdot E_{2}e^{i\phi_{2}}/2$, and $V_{13}=d_{13}\cdot E_{3}e^{i\phi_{3}}/2$ are dipole-interaction matrix elements; ω_12_ = *E*_2_ − *E*_1_ − ω_1_, ω_23_ = *E*_3_ − *E*_2_ − ω_2_, and ω_13_ = *E*_3_ − *E*_1_ − ω_3_ are detunings from exact resonances; and ϕ = ϕ_1_ + ϕ_2_ − ϕ_3_ is the relative TRICC phase. After molecular orientation averaging (see Methods), the energy shift becomes

$$\begin{array}{c} {\langle \delta E\rangle }_{O}=\frac{\text{Re}\;\{(d_{13}^{*}\cdot [d_{12}\times d_{23}])(E_{3}^{*}\cdot [E_{1}\times E_{2}]e^{i\phi })\}}{24\omega_{12}\omega_{13}}\\ +\frac{{|d_{12}|}^{2}{|E_{1}|}^{2}}{12\omega_{12}}+\frac{{|d_{13}|}^{2}{|E_{3}|}^{2}}{12\omega_{13}} \end{array}$$ (3)

The two last terms represent the ordinary Stark shift of the FID-active state (*31*), whereas the first term, the enantiosensitive Stark shift, has an explicit separation into a triple product of molecular dipoles, which carries information about the chirality of the molecule, and a triple product of the driving fields. This term is nonzero if the molecule is chiral and it is driven by an electrically chiral field, and therefore, it provides the enantiosensitivity of the FID-beam steering in our scheme.

The field triple product represents the leading-order nonlinear chiral correlation function of the field (*4*)

$$h^{\left(3\right)}=E_{3}^{*}\cdot (E_{1}\times E_{2})e^{i\phi }$$ (4)

which provides a quantitative measure of the chirality of the Lissajous curve traced out by the TRICC electric field over time. Such a quantitative measure, i.e., a pseudoscalar observable sensitive to the local chirality of the field, does not arise within linear optics-based understandings of optical chirality (*3*). While traditional chiroptical spectroscopy does include nonlinear optical methods (*8*–*11*, *39*) in addition to linear interactions (*3*, *40*), those methods have not required or given rise to a nonlinear measure of optical chirality, which is essential here. In our case, the correct order of nonlinearity is the one dictated by the physics of the TRICC process, a fact reflected in the natural appearance of *h*^(3)^ in the orientation-averaged energy shift (Eq. 3).

### TRICC field

The TRICC field (Eq. 1) itself is a superposition of three components with amplitudes $E_{j}$ and frequencies ω*_j_*. Before the focusing optics that directs the TRICC beams onto the interaction region, the beams are polarized in the plane perpendicular to the propagation direction, with two of these components orthogonal to the third one: E_1_ = {0, E_1_,0}, E_2_ = {E_2_,0,0}, and E_3_ = {E_3_,0,0}. In the near field, each of these components forms a Gaussian focus, which acquires a longitudinal polarization component within the first postparaxial approximation (*37*), giving field amplitudes with spatial dependence of the form

$$\begin{array}{c} E_{1}=i\sqrt{I_{1}}e^{-(x^{2}+y^{2})/w_{1}^{2}}\{0,1,-i\frac{2y}{k_{1}w_{1}^{2}}\},\\ E_{2}=i\sqrt{I_{2}}e^{-(x^{2}+y^{2})/w_{2}^{2}}\{1,0,-i\frac{2x}{k_{2}w_{2}^{2}}\},\\ E_{3}=i\sqrt{I_{3}}e^{-(x^{2}+y^{2})/w_{3}^{2}}\{1,0,-i\frac{2x}{k_{3}w_{3}^{2}}\} \end{array}$$ (5)

at the focal plane *z* = 0, where *I_j_* are field intensities at the center of the focal spot, *k_j_* = ω*_j_*/*c* are wave numbers, and *w_j_* are focal waists.

**Fig. 2.**
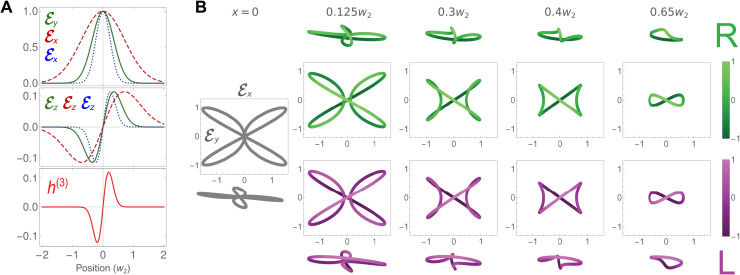
TRICC field. (**A**) Field components of each ω_1_, ω_2_, and ω_3_ TRICC-field color (solid green, dashed red, and dotted blue, respectively) both transverse as a function of *x* (top) and longitudinal as a function of *x* for ω_2_ and ω_3_ and *y* for ω_1_ (middle), producing a nonzero chiral correlation function *h*^(3)^ (bottom). The fields are normalized to the central value of the transverse component. (**B**) 3D Lissajous figures of the TRICC field, forming a “chiral clover,” at different positions along the *x* axis. The first column (gray) shows the achiral case *x* = 0. For *x* ≠ 0, the top two rows (green) correspond to positive values of *x* and the bottom two rows (lilac) to negative values. The two middle rows are the projections of the Lissajous figures on the *xy* plane, and the bottom and top rows show an angled viewpoint. The lightness of the curve (color scales on the right) represents the value of the longitudinal component ℰ*_z_*(*t*). We show fields in a ω_1_ : ω_2_ : ω_3_ = 2 : 1 : 3 configuration with wavelengths λ_1_ = 800 nm, λ_2_ = 1600 nm, and λ_3_ = 533 nm and phases ϕ_1_ = π/3, ϕ_2_ = − π/3, and ϕ_3_ = π, focused to *w_i_* = 1.2λ*_i_* with equal numerical aperture for all three colors. An alternative set of Lissajous figures, showing knotted polarizations (*50*), is included in the Supplementary Materials.

Figure 2A describes the main features of the TRICC field at the focal plane, with the transverse polarization components inducing a longitudinal component, in postparaxial optics, to comply with the Maxwell equations in free space, ∇ · **E** = 0. This longitudinal component provides the three-dimensionality necessary to produce a nonzero chiral correlation function *h*^(3)^, shown in the lower panel.

In the time domain, the three-dimensional (3D) polychromatic field combination (Eq. 1) traces a chiral Lissajous figure over time, shown in Fig. 2B, with opposite chiralities on either side of the optical axis, shown in green (*x* > 0) and lilac (*x* < 0), respectively. These chiral Lissajous figures form enantiomeric pairs: They are exact mirror images of each other, but they cannot be superimposed on each other using only rotations, and as shown in the lower panel of Fig. 2A, they correspond to opposite signs of the chiral correlation function *h*^(3)^. At the center of the beam (*x* = 0), the longitudinal polarization components vanish, leading to a planar Lissajous figure that is therefore achiral.

### FIDLE simulation

To illustrate the mechanics of TRICC enantiosensitive steering more vividly, we now turn to a realistic example. We use as a benchmark the methyloxirane molecule (also denoted as propylene oxide and epoxypropane in strong-field literature; we use the preferred International Union of Pure and Applied Chemistry designation), as shown in Fig. 1C, which is a common choice because of its small size and relative rigidity (*41*). For a realistic molecule, we extend the three-level model system to account for all the relevant transitions, producing a generalization of Eq. 3 discussed in the Supplementary Materials. All of the molecular parameters, including eigenstate energies and transition dipole matrix elements, are obtained from ab initio calculations (see Methods) and are detailed in the Supplementary Materials. The macroscopic parameters are assumed to be similar to those in (*31*, *33*), with a T-shaped nozzle length of ~3 mm and a backing pressure of ~3 bar.

**Fig. 3.**
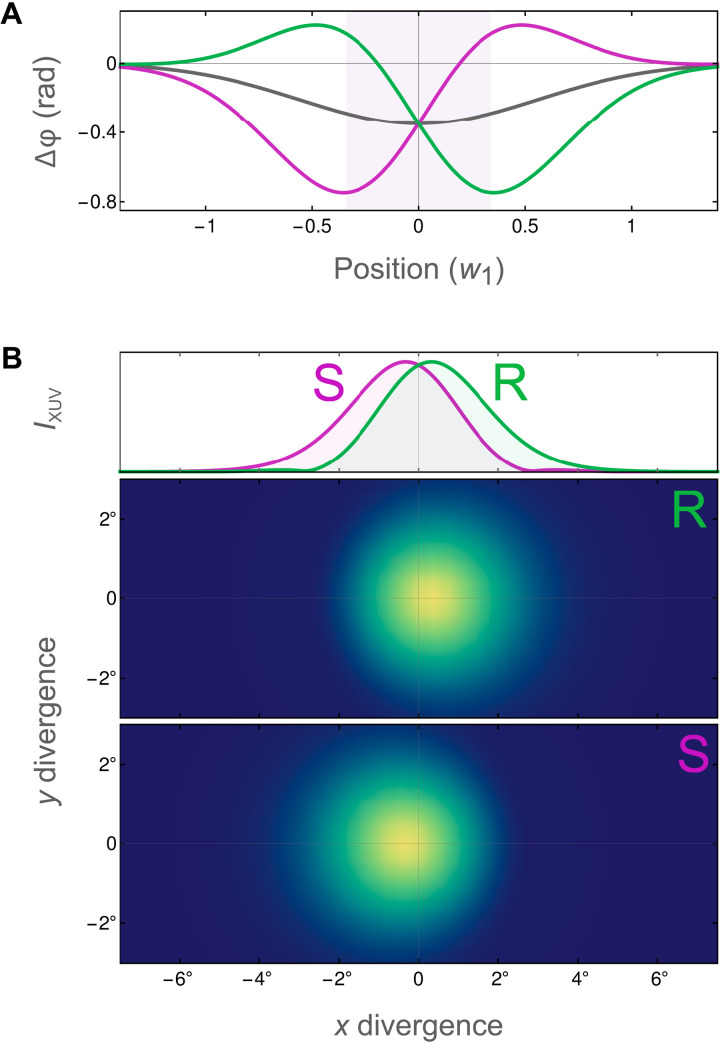
FIDLE by methyloxirane. (**A**) Phase Δφ accumulated in the FID-active Rydberg 3*s* state for R (green) and S (lilac) enantiomers; gray shows an achiral phase. (**B**) FID-beam divergence for each enantiomer, with a lineout of both on the top.

We show in Fig. 3 the enantiosensitive beam steering that results for both near- and far-field observables. For the near field, Fig. 3A displays the quantum phase Δφ induced in the FID-active state of the molecule, for both the S (lilac curve) and R (green curve) enantiomers, as well as for the achiral state (gray curve) obtained from Eq. 3 by removing the chirally sensitive terms. This quantum phase experiences a clear slope over the focal region of the UV beam (shaded in violet), which directly mirrors the chiral nonlinear correlation function *h*^(3)^ of the field and which redirects the beam in opposite directions for different chiralities. This redirection translates into the far-field picture, which we exhibit in Fig. 3B both as a lineout and a 2D image, via a standard spatial Fourier transform. The two beams perform a clearly visible displacement at about half a degree from the initial central position. For a racemic mixture, the opposite phases cancel out, and the beam is not deflected. For an impure but imbalanced mixture, the phase, and therefore the deflection angle, is averaged, providing the basis to use this scheme for measurements of enantiomeric purity.

This result is obtained for a TRICC field at wavelengths λ_1_ = 3438 nm, λ_2_ = 1365 nm, and λ_3_ = 977 nm, chosen to be close to resonances with the Rydberg 3*p* and 3*d* states (see the Supplementary Materials for details and for an alternative choice of wavelengths). We use field intensities *I*_1_ = 2 × 10^10^ W/cm^2^, *I*_2_ = 3.5 × 10^11^ W/cm^2^, and *I*_3_ = 2 × 10^11^ W/cm^2^, phases as in Fig. 2, and a sine-squared envelope starting at *t* = 0 lasting 25 optical cycles (FWHM of amplitude) of the ω_2_ field. The UV beam is focused to a waist of *w*_UV_ = 7λ_UV_ (FWHM), and the TRICC fields are focused to equal waists *w*_1_ = *w*_2_ = *w*_3_ = 1.2λ_1_. These TRICC-field parameters are chosen to minimize population transfer to higher excited states and to keep the TRICC pulse both long enough and with a sufficiently narrow bandwidth, so that it does not trigger any nuclear dynamics that could cause decoherence, but also short enough to enable temporal resolution of the meaningful chiral nuclear-motion modes of the molecule in future time-resolved experiments. We benchmark these results against a direct numerical solution of the TDSE reported in the Supplementary Materials, where we also present detailed ab initio estimations of the role of nuclear motion within the TRICC FIDLE process.

### General steering of FID

**Fig. 4.**
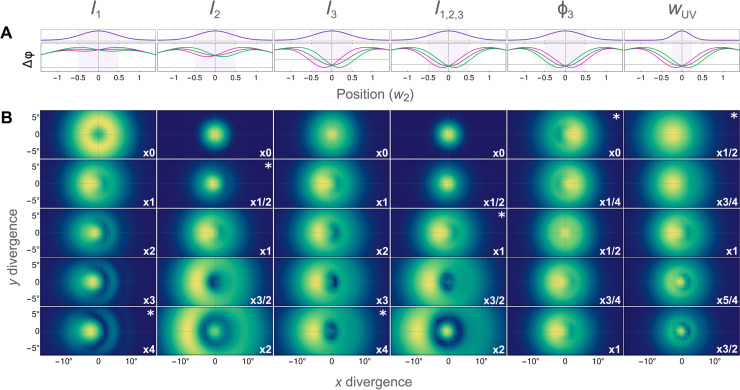
Enantiosensitive FID steering as a function of the TRICC field. Parameter scans reflecting the change in (**A**) the accumulated phase Δφ of the FID-active state and (**B**) the final divergence of the FID beam, as displayed in Fig. 3, for the S enantiomer. The scanned parameter is shown above each column, with asterisks showing the panel corresponding to the phase plot in (A). The overall plot parameters use intensities *I*_1_ = 0.5 × 10^10^ W/cm^2^, *I*_2_ = 8 × 10^11^ W/cm^2^, and *I*_3_ = 1 × 10^11^ W/cm^2^, phase ϕ_3_ = π, and UV focal waist *w*_UV_ = 4λ_UV_. For each column, the scanned parameter is changed via the multiplier at the bottom right of each panel.

To understand more widely how the FIDLE beam steering works, it is also useful to look at broader variations in these parameters. We show this in Fig. 4 for a three-state model of methyloxirane taking only the states closest to resonances with fields of wavelengths λ_1_ = 3.4 μm, λ_2_ = 1.35 μm, and λ_3_ = 966 nm, otherwise using the same parameters as in Fig. 3. The “carpet” in Fig. 4 shows the effect of variations in the driver intensities as well as the relative TRICC-field phase and the UV focal waist.

This carpet demonstrates that the enantiosensitive FID steering is possible at various intensities and with varying degrees of induced structure in the FID beam. We see, in particular, that changing the TRICC-field intensities directly controls the magnitude of the beam divergence angle, whereas the relative phase (given by the ϕ_3_ scan) also controls the direction of the steering. This is natural, because the phase appears in the chiral correlation function *h*^(3)^ defined in Eq. 4, and changing ϕ by π inverts the chirality of the field, and therefore, it inverts its interaction with chiral matter, so the FID beam is redirected in the opposite direction for each fixed enantiomer.

## DISCUSSION

The process that we propose provides for clear enantiosensitive signals from a dilute medium, by harnessing the power of synthetic chiral light while still keeping to a delicate intensity, thereby preserving the molecules largely undisturbed. Our all-optical scheme is general, widely applicable, and compatible with a wide array of ultrafast pump-probe spectroscopies—including multiphoton pumps (*15*, *16*), which would offer background-free FID measurements—and it is built on sources and techniques that are already available. TRICC FIDLE allows the creation of locally chiral light fields that are naturally available within microwave three-wave mixing (*12*) while, at the same time, offering key advantages that are out of reach in the terahertz and gigahertz ranges, including a wider spectral flexibility and, most notably, the ability to couple to electronic and vibronic dynamics. Our work thus opens the door to the design of chiral topological light and its widespread application to chiral spectroscopies in ultrafast science, and it supplies a general tool for optical chiral recognition that can be expanded to allow for triggering and time-resolved probing of chiral molecular dynamics.

## METHODS

### TRICC-driven dynamics of molecular states

We solve the TDSE, using atomic units throughout

$$i\frac{\partial \Psi (t)}{\partial t}=\hat{H}(t)\Psi (t)$$ (6)

for the Hamiltonian $\hat{H}(t)={\hat{H}}_{0}+\hat{V}(t)$, where ${\hat{H}}_{0}$ is the unperturbed Hamiltonian of the molecule ${\hat{H}}_{0}\varphi_{n}=E_{n}\varphi_{n}$, *n* = 1, 2, 3, and *E_n_* values are the energies of the excited states, assuming that the molecule is in the FID-active state ∣1〉 at the start of the TRICC pulse. The term $\hat{V}(t)=-d\cdot E$ describes the interaction of the TRICC laser field (Eq. 1) with the electric field amplitudes **E***_j_*, frequencies ω*_j_*, and phases ϕ*_j_*.

The total wave function Ψ(*t*) is given by

$$\Psi (t)=\sum_{n=1}^{3}c_{n}(t)\varphi_{n}e^{-{\mathrm{iE}}_{n}t}$$ (7)

where *c_n_* values are complex amplitudes of the states ∣1〉, ∣2〉, and ∣3〉 with energies *E_n_*, respectively (see Fig. 1B).

We substitute the total wave function (Eq. 7) into the TDSE (Eq. 6), and by using the standard rotating-wave approximation, we obtain the usual system of differential equations for the complex amplitudes

$$\begin{array}{c} i{\overset{\cdot }{c}}_{1}=-V_{12}e^{-i\omega_{12}t}c_{2}-V_{13}e^{-i\omega_{13}t}c_{3},\\ i{\overset{\cdot }{c}}_{2}=-V_{12}^{*}e^{i\omega_{12}t}c_{1}-V_{23}e^{-i\omega_{23}t}c_{3},\\ i{\overset{\cdot }{c}}_{3}=-V_{13}^{*}e^{i\omega_{13}t}c_{1}-V_{23}^{*}e^{i\omega_{23}t}c_{2} \end{array}$$ (8)

where

$$\begin{array}{c} V_{12}=d_{12}\cdot E_{1}e^{i\phi_{1}}/2,\\ V_{23}=d_{23}\cdot E_{2}e^{i\phi_{2}}/2,\\ V_{13}=d_{13}\cdot E_{3}e^{i\phi_{3}}/2 \end{array}$$ (9)

are interaction matrix elements including the dipole transition matrix elements *d_ij_* = 〈*φ_i_*∣**d**∣*φ_j_*〉 and

$$\begin{array}{c} \omega_{12}=E_{2}-E_{1}-\omega_{1},\\ \omega_{23}=E_{3}-E_{2}-\omega_{2},\\ \omega_{13}=E_{3}-E_{1}-\omega_{3} \end{array}$$ (10)

are detunings from the exact resonance. The solution for the case of the exact resonance of this system is detailed in the corresponding section in the Supplementary Materials.

In the off-resonant case, this system can be solved within perturbation theory (PT) up to the third order, assuming that the main population is concentrated at the lowest excited level ∣1〉. The first step of PT gives

$$\begin{array}{c} i{\overset{\cdot }{c}}_{2}=-V_{12}^{*}e^{i\omega_{12}t}c_{1},\\ i{\overset{\cdot }{c}}_{3}=-V_{13}^{*}e^{i\omega_{13}t}c_{1} \end{array}$$ (11)

or

$$\begin{array}{c} c_{2}=-\frac{V_{12}^{*}}{\omega_{12}}e^{i\omega_{12}t}c_{1},\\ c_{3}=-\frac{V_{13}^{*}}{\omega_{13}}e^{i\omega_{13}t}c_{1} \end{array}$$ (12)

At the second step of PT, we have

$$\begin{array}{c} i{\overset{\cdot }{c}}_{2}=-V_{12}^{*}e^{i\omega_{12}t}c_{1}-\frac{V_{13}^{*}V_{23}}{\omega_{13}}e^{i(\omega_{13}-\omega_{23})t}c_{1},\\ i{\overset{\cdot }{c}}_{3}=-V_{13}^{*}e^{i\omega_{13}t}c_{1}-\frac{V_{12}^{*}V_{23}^{*}}{\omega_{12}}e^{i(\omega_{12}+\omega_{23})t}c_{1} \end{array}$$ (13)

or

$$\begin{array}{c} c_{2}=[\frac{V_{12}^{*}}{\omega_{12}}+\frac{V_{13}^{*}V_{23}}{\omega_{12}\omega_{13}}]e^{i\omega_{12}t}c_{1},\\ c_{3}=[\frac{V_{13}^{*}}{\omega_{13}}+\frac{V_{12}^{*}V_{23}^{*}}{\omega_{12}\omega_{13}}]e^{i\omega_{13}t}c_{1} \end{array}$$ (14)

Last, the third step brings us to the dynamics of the lowest excited state

$$c_{1}(t)=e^{i[\frac{{|V_{12}|}^{2}}{\omega_{12}}+\frac{{|V_{13}|}^{2}}{\omega_{13}}+2\frac{|V_{12}||V_{23}||V_{13}|}{\omega_{12}\omega_{13}}\text{cos}\;\phi ]t}$$ (15)

where ϕ is a relative TRICC phase.

### Orientation averaging

The full optical response is the average of the phase of the emission over all possible molecular orientations, a critical step in comparing the optical response of opposite enantiomers [which is missing in previous related work (*13*)]. For the analytical result in Eq. 15, this can be calculated exactly using the theory of isotropic tensors (*42*).

For the Stark shifts $\frac{{|V_{12}|}^{2}}{\omega_{12}}$ and $\frac{{|V_{13}|}^{2}}{\omega_{13}}$, the orientation-averaged amplitudes simplify to

$${\langle {|V_{12}|}^{2}\rangle }_{O}={\langle (d_{12}\cdot E_{1}e^{i\phi_{1}}/2){\left(d_{12}\cdot E_{1}e^{i\phi_{1}}/2\right)}^{*}\rangle }_{O}$$ (16)

$$=\frac{1}{4}E_{1,i}E_{1,j}^{*}{\langle d_{12,i}d_{12,j}^{*}\rangle }_{O}$$ (17)

using Einstein summations. Here, the orientation average of the molecular tensor $d_{12,i}d_{12,j}^{*}$ reduces it to an isotropic tensor, which must be of the form ${\langle d_{12,i}d_{12,j}^{*}\rangle }_{O}=D_{2}\;\delta_{\mathrm{ij}}$, where the multiplier *D*_2_ is determined by taking the trace

$${3D_{2}=D_{2}\;\delta_{\mathrm{ii}}=\langle {d_{12,i}d_{12,i}^{*}\rangle }_{O}=|d_{12}|}^{2}$$ (18)

and the isotropic δ*_ij_* produces the inner product $\delta_{\mathrm{ij}}\;E_{1,i}E_{1,j}^{*}={|E_{1}|}^{2}$, giving the final result

$${\langle {|V_{12}|}^{2}\rangle }_{O}=\frac{1}{12}|d_{12}{|{}^{2}|E_{1}|}^{2}$$ (19)

For the chirally sensitive triple product, we write

$$|V_{12}||V_{23}||V_{13}|\text{cos}\;\phi =\frac{1}{8}\text{Re}\;[(d_{12}\cdot E_{1})(d_{23}\cdot E_{2})(d_{13}^{*}\cdot E_{3}^{*})e^{i\phi }]$$ (20)

so, similarly to the Stark shifts, we can separate

$$\begin{array}{c} {\langle |V_{12}||V_{23}||V_{13}|\text{cos}\;\phi \rangle }_{O}=\frac{1}{8}\text{Re}\;[E_{1,i}E_{2,j}E_{3,k}^{*}e^{i\phi }\\ {\langle d_{12,i}d_{23,j}d_{13,k}^{*}\rangle }_{O}] \end{array}$$ (21)

Here, the molecular tensor $d_{12,i}d_{23,j}d_{13,k}^{*}$, now of rank 3, averages again to an isotropic tensor, which should be proportional to the Levi-Civita tensor ϵ*_ijk_*, so that ${\langle d_{12,i}d_{23,j}d_{13,k}^{*}\rangle }_{O}=D_{3}\;\epsilon_{\mathrm{ijk}}$, where the multiplier *D*_3_ is found by contracting with a separate Levi-Civita tensor. This gives

$$\begin{array}{c} 6\;D_{3}=D_{3}\;\epsilon_{\mathrm{ijk}}\epsilon_{\mathrm{ijk}}={\langle d_{12,i}d_{23,j}d_{13,k}^{*}\epsilon_{\mathrm{ijk}}\rangle }_{O}\\ =(d_{12}\times d_{23})\cdot d_{13}^{*} \end{array}$$ (22)

in terms of the orientation-invariant scalar triple product of the three dipoles. Last, the resulting factor of ϵ*_ijk_* induces the triple product $E_{1,i}E_{2,j}E_{3,k}^{*}e^{i\phi }\epsilon_{\mathrm{ijk}}=(E_{1}\times E_{2})\cdot E_{3}^{*}e^{i\phi }$ for the fields, giving the final result

$$\begin{array}{c} {\langle |V_{12}||V_{23}||V_{13}|\text{cos}\;\phi \rangle }_{O}=\frac{1}{24}\text{Re}\;[(E_{1}\times E_{2})\cdot E_{3}^{*}e^{i\phi }\\ \left(d_{12}\times d_{23})\cdot d_{13}^{*}\right] \end{array}$$ (23)

### FID phase gradient

We derive a simple expression for the phase gradient (and therefore the beam deflection) produced by the quantum phase $\Delta \varphi =\int_{0}^{2\tau }\delta \mathrm{Edt}$ accumulated by the FID-active state during the TRICC pulse with the energy shift (Eq. 3). Assuming a sine-squared envelope in amplitude on all fields with duration 2τ, the cubic time integral over the pulse boils down to $\int_{0}^{2\tau }{\left({\text{sin}}^{2}(\pi t/2\tau )\right)}^{3}\mathrm{dt}=\frac{5}{8}\tau$. Keeping only the enantiosensitive cubic term, the phase is thus

$${\langle \Delta \varphi_{\text{chiral}}\rangle }_{O}=\frac{5\tau /3}{2^{6}\;\omega_{12}\omega_{13}}\text{Re}\;\{(d_{13}^{*}\cdot [d_{12}\times d_{23}])h^{\left(3\right)}\}$$ (24)

Here, the chiral field correlation function *h*^(3)^ is obtained from the TRICC fields (Eq. 5) and thus reads

$$h^{\left(3\right)}=2x\;e^{-(x^{2}+y^{2})/w^{2}}\sqrt{I_{1}I_{2}I_{3}}(\frac{1}{k_{2}w_{2}^{2}}-\frac{1}{k_{3}w_{3}^{2}})e^{i\phi }$$ (25)

where $w^{-2}=w_{1}^{-2}+w_{2}^{-2}+w_{3}^{-2}$. Under the reasonable assumption that the UV beam waist is smaller than this total TRICC waist, the spatial gradient of *h*^(3)^ corresponds to the linear ramp only and can therefore be approximated as the constant

$$\frac{\partial h^{\left(3\right)}}{\partial x}=2\sqrt{I_{1}I_{2}I_{3}}(\frac{1}{k_{2}w_{2}^{2}}-\frac{1}{k_{3}w_{3}^{2}})e^{i\phi }$$ (26)

This then allows us to get the final phase gradient acquired by the FID radiation, which steers it away from its initial direction and which is given by

$$\begin{array}{c} \frac{\partial }{\partial x}{\langle \Delta \varphi_{\text{chiral}}\rangle }_{O}=\frac{5\tau \sqrt{I_{1}I_{2}I_{3}}}{3\times 2^{5}\;\omega_{12}\omega_{13}}(\frac{1}{k_{2}w_{2}^{2}}-\frac{1}{k_{3}w_{3}^{2}})\\ \times \text{Re}\;\{(d_{13}^{*}\cdot [d_{12}\times d_{23}])e^{i\phi }\} \end{array}$$ (27)

### Ab initio calculation of molecular dipoles

Molecular triple products are calculated for the showcase of the methyloxirane molecule (see table S3). We treat Rydberg states within the multireference configuration interaction with single excitations ansatz. The CAS(2,2) wave function, with the active orbitals localized on the lone pairs of the oxygen atom, was used as the reference. This calculation is performed within the ORMAS (occupation-restricted multiple active space) solver (*43*, *44*) of the GAMESS package (*45*, *46*) using the optimized MP2(fc) (Møller-Plesset to second-order PT with frozen core) method for the geometry shown in table S1.

We use the aug-cc-pVTZ basis set, augmented with several Kaufman-Rydberg functions (with *n* = 1 to 4 and *S*, *P*, *D*, and *F* character) at the center of mass of the molecule to accurately support the Rydberg series (*47*–*49*). The energies of the eigenstates of interest (discarding spin triplet states) are reported in detail in the Supplementary Materials.

## Supplementary Materials

### This PDF file includes:

TRICC-field configuration Ab initio calculations for methyloxirane molecule Analysis of the contribution of nuclear motion Benchmarking of TRICC dynamics in methyloxirane Additional results Resonant case Figs. S1 to S6 Tables S1 to S6 References

## References

[1] G. Palyi, *Biological Chirality* (Academic Press, 2019).

[2] L. Pasteur, *Researches on the molecular asymmetry of natural organic products* (Alembic Club, 1905); ark:/13960/t77t0rb8m [translation from *Recherches sur la dissymétrie moléculaire des produits organiques naturels* (1861)].

[3] Y. Tang, A. E. Cohen, Optical chirality and its interaction with matter. Phys. Rev. Lett. 104, 163901 (2010). 20482049 10.1103/PhysRevLett.104.163901

[4] D. Ayuso, O. Neufeld, A. F. Ordonez, P. Decleva, G. Lerner, O. Cohen, M. Ivanov, O. Smirnova, Synthetic chiral light for efficient control of chiral light–matter interaction. Nat. Photonics 13, 866–871 (2019).

[5] O. Neufeld, D. Ayuso, P. Decleva, M. Y. Ivanov, O. Smirnova, O. Cohen, Ultrasensitive chiral spectroscopy by dynamical symmetry breaking in high harmonic generation. Phys. Rev. X 9, 031002 (2019).

[6] D. Ayuso, A. F. Ordonez, M. Ivanov, O. Smirnova, Ultrafast optical rotation in chiral molecules with ultrashort and tightly focused beams. Optica 8, 1243–1246 (2021).

[7] A. F. Ordonez, O. Smirnova, Generalized perspective on chiral measurements without magnetic interactions. Phys. Rev. A 98, 063428 (2018).

[8] P. M. Rentzepis, J. A. Giordmaine, K. W. Wecht, Coherent optical mixing in optically active liquids. Phys. Rev. Lett. 16, 792–794 (1966).

[9] P. Fischer, D. S. Wiersma, R. Righini, B. Champagne, A. D. Buckingham, Three-wave mixing in chiral liquids. Phys. Rev. Lett. 85, 4253–4256 (2000). 11060611 10.1103/PhysRevLett.85.4253

[10] G. J. Simpson, Molecular origins of the remarkable chiral sensitivity of second-order nonlinear optics. Chemphyschem 5, 1301–1310 (2004). 15499846 10.1002/cphc.200300959

[11] M. A. Belkin, Y. R. Shen, Non-linear optical spectroscopy as a novel probe for molecular chirality. Int. Rev. Phys. Chem. 24, 257–299 (2005).

[12] D. Patterson, M. Schnell, J. M. Doyle, Enantiomer-specific detection of chiral molecules via microwave spectroscopy. Nature 497, 475–477 (2013). 23698447 10.1038/nature12150

[13] Y.-Y. Chen, C. Ye, Q. Zhang, Y. Li, Enantio-discrimination via light deflection effect. J. Chem. Phys. 152, 204305 (2020). 32486668 10.1063/5.0008157

[14] S. Eibenberger, J. Doyle, D. Patterson, Enantiomer-specific state transfer of chiral molecules. Phys. Rev. Lett. 118, 123002 (2017). 28388207 10.1103/PhysRevLett.118.123002

[15] C. Lux, M. Wollenhaupt, T. Bolze, Q. Liang, J. Köhler, C. Sarpe, T. Baumert, Circular dichroism in the photoelectron angular distributions of camphor and fenchone from multiphoton ionization with femtosecond laser pulses. Angew. Chem. Int. Ed. 51, 5001–5005 (2012). 10.1002/anie.201109035 22351439

[16] C. S. Lehmann, N. B. Ram, I. Powis, M. H. M. Janssen, Imaging photoelectron circular dichroism of chiral molecules by femtosecond multiphoton coincidence detection. J. Chem. Phys. 139, 234307 (2013). 24359367 10.1063/1.4844295

[17] S. Beaulieu, A. Comby, D. Descamps, B. Fabre, G. A. Garcia, R. Géneaux, A. G. Harvey, F. Légaré, Z. Mašín, L. Nahon, A. F. Ordonez, S. Petit, B. Pons, Y. Mairesse, O. Smirnova, V. Blanchet, Photoexcitation circular dichroism in chiral molecules. Nat. Phys. 14, 484–489 (2018).

[18] A. F. Ordonez, O. Smirnova, Propensity rules in photoelectron circular dichroism in chiral molecules. II. General picture. Phys. Rev. A 99, 043417 (2019). 10.1039/d1cp05485f 35188152

[19] R. Cireasa, A. E. Boguslavskiy, B. Pons, M. C. H. Wong, D. Descamps, S. Petit, H. Ruf, N. Thiré, A. Ferré, J. Suarez, J. Higuet, B. E. Schmidt, A. F. Alharbi, F. Légaré, V. Blanchet, B. Fabre, S. Patchkovskii, O. Smirnova, Y. Mairesse, V. R. Bhardwaj, Probing molecular chirality on a sub-femtosecond timescale. Nat. Phys. 11, 654–658 (2015).

[20] M. Pitzer, M. Kunitski, A. S. Johnson, T. Jahnke, H. Sann, F. Sturm, L. P. H. Schmidt, H. Schmidt-Böcking, R. Dörner, J. Stohner, J. Kiedrowski, M. Reggelin, S. Marquardt, A. Schießer, R. Berger, M. S. Schöffler, Direct determination of absolute molecular stereochemistry in gas phase by Coulomb explosion imaging. Science 341, 1096–1100 (2013). 24009390 10.1126/science.1240362

[21] C. Pérez, A. L. Steber, S. R. Domingos, A. Krin, D. Schmitz, M. Schnell, Coherent enantiomer-selective population enrichment using tailored microwave fields. Angew. Chem. Int. Ed. 56, 12512–12517 (2017). 10.1002/anie.201704901 28672055

[22] O. Neufeld, H. Hübener, A. Rubio, U. De Giovannini, Strong chiral dichroism and enantiopurification in above-threshold ionization with locally chiral light. Phys. Rev. Res. 3, L032006 (2021).

[23] F. Krausz, M. Ivanov, Attosecond physics. Rev. Mod. Phys. 81, 163–234 (2009).

[24] D. M. Villeneuve, Attosecond science. Contemp. Phys. 59, 47–61 (2018).

[25] J. Biegert, F. Calegari, N. Dudovich, F. Quéré, M. Vrakking, Attosecond technology(ies) and science. J. Phys. B. At. Mol. Opt. Phys. 54, 070201 (2021).

[26] S. Mukamel, *Principles of Nonlinear Optical Spectroscopy* (Oxford Univ. Press, 1995).

[27] H. Rubinsztein-Dunlop, A. Forbes, M. V. Berry, M. R. Dennis, D. L. Andrews, M. Mansuripur, C. Denz, C. Alpmann, P. Banzer, T. Bauer, E. Karimi, L. Marrucci, M. Padgett, M. Ritsch-Marte, N. M. Litchinitser, N. P. Bigelow, C. Rosales-Guzmán, A. Belmonte, J. P. Torres, T. W. Neely, M. Baker, R. Gordon, A. B. Stilgoe, J. Romero, A. G. White, R. Fickler, A. E. Willner, G. Xie, B. McMorran, A. M. Weiner, Roadmap on structured light. J. Opt. 19, 013001 (2016).

[28] M. Chini, X. Wang, Y. Cheng, H. Wang, Y. Wu, E. Cunningham, P.-C. Li, J. Heslar, D. A. Telnov, S.-I. Chu, Z. Chang, Coherent phase-matched VUV generation by field-controlled bound states. Nat. Photonics 8, 437–441 (2014).

[29] S. Beaulieu, E. Bloch, L. Barreau, A. Comby, D. Descamps, R. Géneaux, F. Légaré, S. Petit, Y. Mairesse, Phase-resolved two-dimensional spectroscopy of electronic wave packets by laser-induced XUV free induction decay. Phys. Rev. A 95, 041401 (2017).

[30] H. Yun, J. H. Mun, S. I. Hwang, S. B. Park, I. A. Ivanov, C. H. Nam, K. T. Kim, Coherent extreme-ultraviolet emission generated through frustrated tunnelling ionization. Nat. Photonics 12, 620–624 (2018).

[31] S. Bengtsson, E. W. Larsen, D. Kroon, S. Camp, M. Miranda, C. L. Arnold, A. L’Huillier, K. J. Schafer, M. B. Gaarde, L. Rippe, J. Mauritsson, Space–time control of free induction decay in the extreme ultraviolet. Nat. Photonics 11, 252–258 (2017).

[32] L. Drescher, O. Kornilov, T. Witting, G. Reitsma, N. Monserud, A. Rouzée, J. Mikosch, M. J. J. Vrakking, B. Schütte, Extreme-ultraviolet refractive optics. Nature 564, 91–94 (2018). 30487603 10.1038/s41586-018-0737-3

[33] S. Bengtsson, J. Mauritsson, Ultrafast control and opto-optical modulation of extreme ultraviolet light. J. Phys. B: At. Mol. Opt. Phys. 52, 063002 (2019).

[34] L. Drescher, O. Kornilov, T. Witting, V. Shokeen, M. J. J. Vrakking, B. Schütte, Extreme-ultraviolet spectral compression by four-wave mixing. Nat. Photonics 15, 263–266 (2021).

[35] A. Olofsson, E. R. Simpson, N. Ibrakovic, S. Bengtsson, J. Mauritsson, Spatial control of extreme ultraviolet light with opto-optical phase modulation. Opt. Lett. 46, 2356–2359 (2021). 33988582 10.1364/OL.422049

[36] S. Ghosh, G. Herink, A. Perri, F. Preda, C. Manzoni, D. Polli, G. Cerullo, Broadband optical activity spectroscopy with interferometric Fourier-transform balanced detection. ACS Photonics 8, 2234–2242 (2021). 34476287 10.1021/acsphotonics.0c01866 PMC8377715

[37] K. Y. Bliokh, F. Nori, Transverse and longitudinal angular momenta of light. Phys. Rep. 592, 1–38 (2015).

[38] K. Y. Bliokh, F. J. Rodríguez-Fortuño, F. Nori, A. V. Zayats, Spin-orbit interactions of light. Nat. Photonics 9, 796–808 (2015).

[39] L. D. Barron, L. Hecht, I. H. McColl, E. W. Blanch, Raman optical activity comes of age. Mol. Phys. 102, 731–744 (2004).

[40] A. Salam, W. J. Meath, On enantiomeric excesses obtained from racemic mixtures by using circularly polarized pulsed lasers of varying durations. Chem. Phys. 228, 115–129 (1998).

[41] V. Barone, M. Biczysko, J. Bloino, C. Puzzarini, Accurate molecular structures and infrared spectra of trans-2,3-dideuterooxirane, methyloxirane, and trans-2,3-dimethyloxirane. J. Chem. Phys. 141, 034107 (2014). 25053301 10.1063/1.4887357 PMC4612369

[42] D. L. Andrews, T. Thirunamachandran, On three-dimensional rotational averages. J. Chem. Phys. 67, 5026–5033 (1977).

[43] J. Ivanic, Direct configuration interaction and multiconfigurational self-consistent-field method for multiple active spaces with variable occupations. I. Method. J. Chem. Phys. 119, 9364–9376 (2003).

[44] J. Ivanic, Direct configuration interaction and multiconfigurational self-consistent-field method for multiple active spaces with variable occupations. II. Application to oxoMn(salen) and N_2_O_4_. J. Chem. Phys. 119, 9377–9385 (2003).

[45] M. W. Schmidt, K. K. Baldridge, J. A. Boatz, S. T. Elbert, M. S. Gordon, J. H. Jensen, S. Koseki, N. Matsunaga, K. A. Nguyen, S. Su, T. L. Windus, M. Dupuis, J. A. Montgomery, General atomic and molecular electronic structure system. J. Comput. Chem. 14, 1347–1363 (1993).

[46] C. Dykstra, *Theory and Applications of Computational Chemistry: The First Forty Years* (Elsevier, 2005).

[47] T. H. Dunning, Gaussian basis sets for use in correlated molecular calculations. I. The atoms boron through neon and hydrogen. J. Chem. Phys. 90, 1007–1023 (1989).

[48] R. A. Kendall, T. H. Dunning Jr., R. J. Harrison, Electron affinities of the first-row atoms revisited. Systematic basis sets and wave functions. J. Chem. Phys. 96, 6796–6806 (1992).

[49] K. Kaufmann, W. Baumeister, M. Jungen, Universal Gaussian basis sets for an optimum representation of Rydberg and continuum wavefunctions. J. Phys. B. At. Mol. Opt. Phys. 22, 2223–2240 (1989).

[50] D. Sugic, M. R. Dennis, F. Nori, K. Y. Bliokh, Knotted polarizations and spin in three-dimensional polychromatic waves. Phys. Rev. Res. 2, 042045 (2020).

[51] C. Adams, *The Knot Book: An Elementary Introduction to the Mathematical Theory of Knots* (American Mathematical Society, 2004).

[52] K. Kimura, S. Katsumata, Y. Achiba, T. Yamazaki, S. Iwata, *Handbook of HeI Photoelectron Spectra of Fundamental Organic Molecules. Ionization Energies, Ab Initio Assignments, and Valence Electronic Structure for 200 Molecules* (Halstead Press, 1981).

[53] J. H. Hannay, J. F. Nye, Fibonacci numerical integration on a sphere. J. Phys. A Math. Gen. 37, 11591–11601 (2004).

[54] Yarchik, https://mathematica.stackexchange.com/users/9469/yarchik, Package for fast spherical harmonic transform in Mathematica? [retrieved 17 September 2021]; https://mathematica.stackexchange.com/a/171856.

