# Supplementary material for: Enantiosensitive steering of free-induction decay
Source: Sci Adv. 2022 Jun 15;8(24):eabq1962. doi: 10.1126/sciadv.abq1962 (PMC9200270; doi:10.1126/sciadv.abq1962)
Supplement: Supplementary file 1 — TRICC-field configuration Ab initio calculations for methyloxirane molecule Analysis of the contribution of nuclear motion Benchmarking of TRICC dynamics in methyloxirane Additional results Resonant case Figs. S1 to S6 Tables S1 to S6 References [file sciadv.abq1962_sm.pdf]

Science Advances
AAAS

Supplementary Materials for

Enantiosensitive steering of free-induction decay

Margarita Khokhlova *et al.*

Corresponding author: Margarita Khokhlova, m.khokhlova@imperial.ac.uk

Sci. Adv. **8**, eabq1962 (2022)
DOI: 10.1126/sciadv.abq1962

This PDF file includes:

TRICC-field configuration
Ab initio calculations for methyloxirane molecule
Analysis of the contribution of nuclear motion
Benchmarking of TRICC dynamics in methyloxirane
Additional results
Resonant case
Figs. S1 to S6
Tables S1 to S6
References

TRICC-field configuration

Here we present an alternative configuration of the TRICC field, shown in Figure S1 using identical conventions to Figure 2 of the main text. One can observe here that upon moving from the achiral middle of the beam, the Lissajous figure first acquires a complex knotted structure [50], which then unknots itself between $x = 0.3w_2$ and $x = 0.4w_2$, followed by shrinking in size as a structure, corresponding to a decrease in intensity. This behaviour appears symmetrically in both directions from $x = 0$, but with opposite chirality. The knot here is isomorphic to the trefoil knot, which is the simplest possible chiral knot [51].

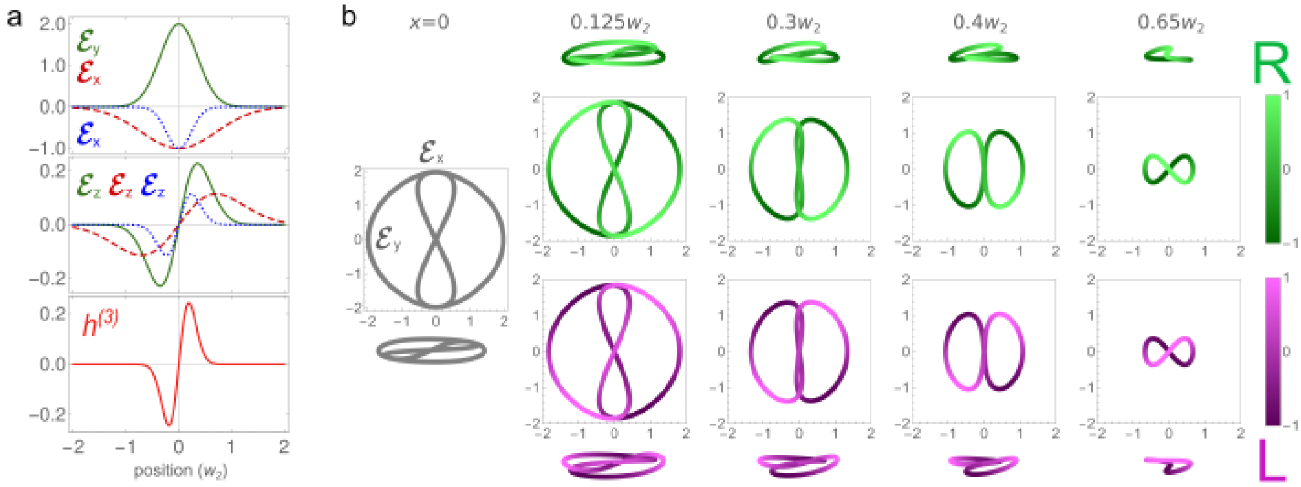

Figure S1 | Alternative TRICC field. (a) Field components of each ω_1, ω_2 and ω_3 TRICC-field colour (solid green, dashed red and dotted blue, respectively) both transverse as a function of x (top) and longitudinal as a function of x for ω_2, ω_3 and y for ω_1 (middle), producing a nonzero chiral correlation function $h^{(3)}$ (bottom). The fields are normalised to the central value of the transverse component. (b) 3D Lissajous figures of the TRICC field, forming a 'chiral clover', at different positions along the x axis. The first column (gray) shows the achiral case $x = 0$. For $x \neq 0$, the top two rows (green) correspond to positive values of x and the bottom two rows (lilac) to negative values. The middle two rows are the projections of the Lissajous figures on the xy-plane, and bottom and top show an angled viewpoint. The lightness of the curve (colour scales on the right) represents the value of the longitudinal component $\mathcal{E}_z(t)$. We show fields in a $\omega_1{:}\omega_2{:}\omega_3 = 2{:}1{:}3$ configuration with wavelengths $\lambda_1 = 800\,\mathrm{nm}$, $\lambda_2 = 1600\,\mathrm{nm}$ and $\lambda_3 = 533\,\mathrm{nm}$ and phases $\phi_1 = 0$, $\phi_2 = \pi/2$ and $\phi_3 = \pi/2$ and intensity ratio $I_1{:}I_2{:}I_3 = 4{:}1{:}1$, focused to $w_i = 1.2\lambda_i$ with equal numerical aperture for all three colours.

Ab initio calculations for methyloxirane molecule

Molecular triple products are calculated for the methyloxirane molecule. This calculation is performed within the ORMAS solver of the GAMESS package using the optimised MP2(fc), for the geometry shown in Table S1.

We use the aug-cc-pVTZ basis set, augmented with several Kaufman-Rydberg functions (with $n = 1$ through 4 and S, P, D and F character) at the centre of mass of the molecule to accurately support the Rydberg series of the molecule. The energies of the eigenstates of interest (discarding spin triplet states) are reported in detail in Table S2. These are in broad agreement with previous numerical and experimental results [41]. The molecular orbitals corresponding to the excited states from this list are shown in Figure S2.

The calculated transition dipoles $\mathbf{d}_{i,j}$ for all states under consideration are given in Table S3. Excitation to all of these

Atom	x-coordinate	y-coordinate	z-coordinate
C	-0.211761865044	-0.051130800700	0.491070227321
C	0.952135220773	-0.694687579808	-0.118609052200
C	-1.548826197176	-0.044575312951	-0.182512567928
O	0.813397556144	0.738851113898	-0.138422794998
H	1.756284129428	-1.062783697723	0.505504521687
H	0.836773710857	-1.171275968924	-1.084528278135
H	-0.214501788525	0.043535717665	1.572219470170
H	-2.072448641908	0.889377235478	0.018740574166
H	-2.163990948087	-0.867454009803	0.182557270784
H	-1.425212839981	-0.146417011606	-1.259309512207

Table S1 | Molecular geometry of S-methyloxirane. For the R enantiomer, we spatially invert ($\mathbf{r} \mapsto -\mathbf{r}$) all atomic positions.

n	type	E [eV]
1	ground	0.000
3	$3s$	7.279
5	$3p_y$	7.647
7	$3p_z$	7.676
9	$3p_x$	7.846
12	$3d_{z^2-x^2}$	8.452
13	$3d_{z^2-y^2}$	8.468
16	$3d_{xz}$	8.492
17	$3d_{yz}$	8.517
19	$3d_{xy}$	8.539

Table S2 | Excitation energies of neutral methyloxirane.

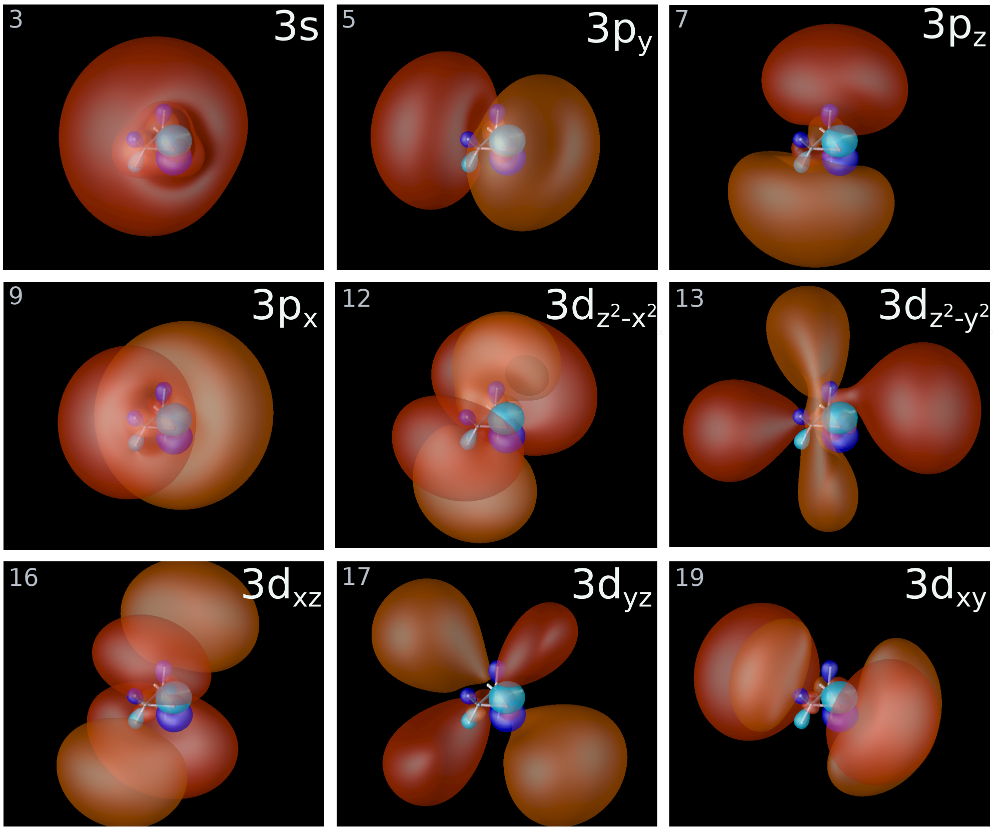

Figure S2 | Molecular orbitals of Rydberg states of methyloxirane. The state indices marked on the top-left corner of each diagram correspond to the index n in Table S2.

states is dipole-allowed as expected for a C_1 molecule. For added clarity, we also present the vector triple products of transition dipoles between $3s$, $3p$ and $3d$ states. In Table S4 we show the major values of these triple-dipole products.

Analysis of the contribution of nuclear motion

In this section we investigate the possible contribution of nuclear motion to the TRICC FIDLE dynamics. The framework we present in this work focuses on electronic motion, and as such, nuclear motion carries a risk of decoherence. We present *ab initio* evidence that this decoherence is not a danger for our parameters.

There are two contributions we need to be concerned with. The first contribution is vibrational excitation due to the geometry change upon Rydberg-state preparation by the UV/XUV pulse. We examine this effect by calculating the harmonic Franck-Condon factors. Within this setting, nuclear dynamics remains negligible so long as the bandwidth of the pre-exciting pulse is below the separation between the desired 0-0 transition and nearest strong vibrational lines. We present the principal calculated Franck-Condon factors in Table S5, showing the overlap of the ground-state nuclear wavepacket to the various nuclear-excitation modes of the methyloxirane cation, which is a good substitute for the Rydberg-excited states as regards the nuclear motion. Our results agree reasonably well with the low-energy part of the experimental spectrum obtained from standard reference data [52].

As one can see, the nearest significantly-active mode is no. 10, at 536 cm^{-1}. As long as the UV pre-excitation pulse has a bandwidth below 60 meV (corresponding to a pulse duration of more than 6 fs), it addresses the 0-0 transition individually,

$\mathbf{d}_{i,j}$	x-component	y-component	z-component
$\mathbf{d}_{1,1}$	-2.657525769894	-1.543353440217	-0.228036682970
$\mathbf{d}_{1,3}$	0.091988977246	0.098002456237	-0.095712584970
$\mathbf{d}_{1,5}$	-0.114785379164	0.004980558892	-0.289690684997
$\mathbf{d}_{1,7}$	0.037337506874	0.212272863307	-0.184052734825
$\mathbf{d}_{1,9}$	-0.164384462658	0.121154631329	-0.053858352076
$\mathbf{d}_{1,12}$	0.070286207770	0.069626845409	0.037556561082
$\mathbf{d}_{1,13}$	-0.074199408815	-0.013384363110	0.010511482901
$\mathbf{d}_{1,16}$	0.027297040930	0.092264353832	0.103006395326
$\mathbf{d}_{1,17}$	0.057685396468	0.042479894136	-0.040279222614
$\mathbf{d}_{1,19}$	0.095843990557	0.054546484613	0.070448835820
$\mathbf{d}_{3,3}$	-1.448749800607	-2.470471844118	1.249356231590
$\mathbf{d}_{3,5}$	-1.114511350645	-3.947903746065	0.334427377246
$\mathbf{d}_{3,7}$	-0.558991208987	0.809582255126	4.517508937859
$\mathbf{d}_{3,9}$	4.114951108384	-1.182732295471	0.590975182563
$\mathbf{d}_{3,12}$	0.168949478830	-0.350672741671	0.037572073151
$\mathbf{d}_{3,13}$	0.446935313569	0.483329506287	-0.373947285238
$\mathbf{d}_{3,16}$	0.363094365047	0.003900424116	0.164417081654
$\mathbf{d}_{3,17}$	-0.038559865609	0.021972944780	-0.104292850068
$\mathbf{d}_{3,19}$	-0.092595456686	0.055480939162	-0.202647782194
$\mathbf{d}_{5,5}$	-2.180684792012	-2.968182981068	0.101798958996
$\mathbf{d}_{5,7}$	0.254188557147	0.480999249790	-0.253778457339
$\mathbf{d}_{5,9}$	-0.104381185028	0.986303830685	-0.007190547362
$\mathbf{d}_{5,12}$	-0.711881012677	-0.084504239273	0.625397520615
$\mathbf{d}_{5,13}$	1.533777013099	-3.101539229162	-1.246498312830
$\mathbf{d}_{5,16}$	-0.551530989191	-0.240930033441	-0.536682494495
$\mathbf{d}_{5,17}$	-0.378927665922	1.268416459796	-3.314499363107
$\mathbf{d}_{5,19}$	-2.848550143508	-2.313210974351	-0.012329313169
$\mathbf{d}_{7,7}$	-2.328870334263	-2.285466458866	-1.270255263971
$\mathbf{d}_{7,9}$	-0.509338021447	-0.098476829478	-1.107049326475
$\mathbf{d}_{7,12}$	-1.237545945677	-0.541292624163	-2.233025481185
$\mathbf{d}_{7,13}$	0.137385765355	1.504056651851	-2.133313540053
$\mathbf{d}_{7,16}$	3.605808682847	-0.692177595294	-1.260955079547
$\mathbf{d}_{7,17}$	0.208138130879	3.323895563713	0.458193512005
$\mathbf{d}_{7,19}$	-0.354540262592	0.022282934444	-1.301367228212
$\mathbf{d}_{9,9}$	-3.874288814724	-1.550375724064	0.058365661961
$\mathbf{d}_{9,12}$	4.935063470291	-0.657198772570	-1.076021479885
$\mathbf{d}_{9,13}$	-0.535928496260	-2.796788649119	-0.201588961480
$\mathbf{d}_{9,16}$	1.045256445603	0.880354995134	3.983950035917
$\mathbf{d}_{9,17}$	1.150385445327	1.263419868559	-0.768887170659
$\mathbf{d}_{9,19}$	-1.465435303259	3.108773778035	-1.192956486852
$\mathbf{d}_{12,12}$	-5.089175393465	-2.443470348450	-0.298934325837
$\mathbf{d}_{12,13}$	1.623607969100	0.301040670738	-0.800995671608
$\mathbf{d}_{12,16}$	-0.758543852920	-0.045759323038	-0.562841534292
$\mathbf{d}_{12,17}$	-0.810318121435	0.327913759159	-0.144181702084
$\mathbf{d}_{12,19}$	0.324395698475	-1.135479304243	-0.053931325799
$\mathbf{d}_{13,13}$	-4.182746411120	-1.014375241188	0.905380972497
$\mathbf{d}_{13,16}$	0.156653112160	0.175851142115	0.927768518583
$\mathbf{d}_{13,17}$	0.620771731324	-1.104818811914	0.928724046325
$\mathbf{d}_{13,19}$	1.053932021966	0.598515893605	-0.356018369896
$\mathbf{d}_{16,16}$	-4.295792962226	-2.312435677284	-0.630725463029
$\mathbf{d}_{16,17}$	-0.543105275103	-0.794254672959	-0.373800186865
$\mathbf{d}_{16,19}$	0.158664049166	-0.502310230162	0.770209037724
$\mathbf{d}_{17,17}$	-3.688302409687	-1.917649666414	-1.585518036329
$\mathbf{d}_{17,19}$	-0.569701898997	-0.370546016532	-0.502881920693
$\mathbf{d}_{19,19}$	-4.497206718969	-2.156411622373	-0.418743285228

Table S3 | Transition dipole moments. Calculated values of transition dipole moment components, in atomic units.

	state	12 $3d_{z^2-x^2}$	13 $3d_{z^2-y^2}$	16 $3d_{xz}$	17 $3d_{yz}$	19 $3d_{xy}$
5	$3p_y$	-0.675	-1.318	0.482	-0.269	1.627
7	$3p_z$	2.555	-3.729	0.409	0.802	-0.078
9	$3p_x$	-2.177	5.634	-1.162	-0.606	-1.977

Table S4 | Values of molecular triple-dipole products. The lowest $3s$ state is common for each product.

Relative intensity	Energy (cm^{-1})	Energy (eV)	Mode	
1.000	0.0	0.000	0	
0.046	355.7	0.044	9	-CH_3 out-of-plane wag
0.198	536.3	0.066	10	(CH_3)CH-O in-plane stretch
0.106	734.2	0.091	11	CH_2-O in-plane stretch
0.046	816.4	0.101	12	-H out-of-plane wag
0.051	1044.0	0.129	15	-H out-of-plane wag
0.211	1139.5	0.141	16	-H (CH) out-of-plane wag
0.298	1177.5	0.146	17	-CH_2 in-plane wag
0.131	1204.0	0.149	18	-H wag
0.133	1269.4	0.157	19	-H (CH) wag
0.050	1713.8	0.212	10,17	
0.065	2317.0	0.287	16,17	
0.047	2355.0	0.292	17,17	
0.040	2381.5	0.295	17,18	

Table S5 | Franck-Condon factors. Franck-Condon factors for the excitation from the neutral ground state to the first excited Rydberg state.

without exciting any vibrational motion which could cause dephasing.

The second contribution we need to consider here is vibrational excitation due to impulsive-Raman transitions caused by the TRICC control pulse. Excitation in either the ground or the Rydberg state affects the coherence, and therefore the FID signal. Here it is sufficient to estimate the maximum excitation amplitude starting from the zeroth vibrational state on both the neutral and cationic potential energy surfaces, where we use the cation as a suitable substitute for the excited Rydberg states as above.

For this mechanism, the driver frequencies are far off-resonance with the vibrational modes. It is therefore sufficient to consider the impulsive-Raman excitation amplitudes, starting from the ground vibrational state. For situations where the IR pulse duration is comparable to (or longer than) the vibrational period, this will over-estimate the excitation amplitude. For simplicity, we consider only the strongest control field – the 1365 nm field (which has 3.5×10^{11} $\mathrm{W/cm^2}$ peak intensity, a sine-squared envelope, and duration of 25 optical cycles).

We present in Table S6 the calculated amplitudes for impulsive-Raman excitation to different vibrational modes of both the neutral and the cation, as well as the oscillation frequencies of those modes, under these conditions. Here the excitation amplitudes are absolute, so the corresponding populations are given by the square of the amplitudes; the sign of the amplitude reflects the initial direction of motion. None of the calculated excitation amplitudes exceeds 0.01, corresponding to populations below 10^{-4} for all of the vibrational modes. It is therefore safe to neglect vibrational excitation by the TRICC control field itself.

To summarise, then, vibrational nuclear motion can be neglected within our scheme: both for the control field, which is not strong enough to produce meaningful impulsive-Raman excitations, as well as for the pre-exciting UV pump pulse, which must be longer than 6 fs to avoid Franck-Condon excitations.

Benchmarking of TRICC dynamics in methyloxirane

To understand how visible the enantiosensitive FID effect can be in the ‘real’ system, we consider two schemes shown in Figure S3, where all the states with their energies and transition dipoles are taken from the *ab-initio* calculations.

The two schemes differ from each other by the choice of which $3p$ state is closest to the resonance (either the lowest, $3p_y$, or the highest, $3p_x$), but with the same $3d$ state, $3d_{xy}$, being closest to the resonance. These two schemes realise situations when values of both (i) the triple molecular-dipole product and (ii) the triple TRICC-field product are as large as possible, but still ensure that the molecule has population left in the FID-active state ($|1\rangle$ or here $3s$) by the end of the TRICC pulse.

Figure S4 shows the numerically-calculated population of the FID-active $3s$ state during the TRICC-field pulse for different orientations of the molecule, for both schemes shown in Figure S3. Here one can see that the majority of orientations presents relatively high population of the $3s$ state after the pulse, while a minority ends up with down to (a) 20% and (b) 5% population, which still secures the final effect. These simulations are obtained by direct solution of the TDSE (see Methods) in the full system of states listed in Table S2 with the dipole moments listed in Table S3, using the standard numerical ODE integration functions of the MATHEMATICA software package.

Mode	Frequency (cm^{-1})		Excitation amplitude	
	neutral	cation	neutral	cation
1	3245.13	3240.63	9.92589×10^{-4}	1.12292×10^{-3}
2	3179.26	3209.99	2.91575×10^{-3}	1.38714×10^{-3}
3	3161.53	3182.28	-1.15214×10^{-3}	2.97432×10^{-3}
4	3159.49	3174.55	4.38974×10^{-3}	3.25967×10^{-3}
5	3142.74	3131.43	-6.07098×10^{-3}	-5.92562×10^{-3}
6	3072.47	3082.45	-8.64631×10^{-3}	-8.30023×10^{-3}
7	1536.54	1506.07	-1.15665×10^{-3}	-4.14146×10^{-5}
8	1514.19	1491.63	-1.39732×10^{-4}	-1.66794×10^{-5}
9	1499.22	1466.90	6.60753×10^{-5}	3.48907×10^{-4}
10	1450.62	1415.58	-1.63081×10^{-3}	-1.63760×10^{-4}
11	1404.12	1398.43	8.40753×10^{-4}	-1.00080×10^{-3}
12	1295.89	1269.63	-3.29044×10^{-3}	-2.22400×10^{-3}
13	1193.89	1204.28	1.96717×10^{-3}	1.80815×10^{-3}
14	1170.87	1177.67	-9.94288×10^{-5}	5.48400×10^{-4}
15	1154.16	1139.74	6.36291×10^{-4}	-2.52131×10^{-4}
16	1127.23	1044.15	-8.11058×10^{-4}	-2.10396×10^{-3}
17	1048.03	944.37	-1.07138×10^{-3}	-2.60184×10^{-3}
18	976.95	927.32	-2.73679×10^{-3}	7.86130×10^{-4}
19	909.98	816.53	-8.89245×10^{-4}	-1.25882×10^{-3}
20	858.53	734.38	-7.63578×10^{-4}	-7.53430×10^{-4}
21	776.28	536.49	1.34914×10^{-3}	-2.77898×10^{-4}
22	405.77	355.79	-1.23773×10^{-3}	-1.31225×10^{-3}
23	368.53	322.08	5.09989×10^{-4}	7.76622×10^{-5}
24	217.91	202.58	-1.80736×10^{-4}	-1.24175×10^{-4}

Table S6 | Vibrational excitation amplitudes. Excitation amplitudes into different modes of both the neutral and the cation, together with the vibrational frequencies of those modes, for impulsive-Raman excitation caused by the TRICC control field.

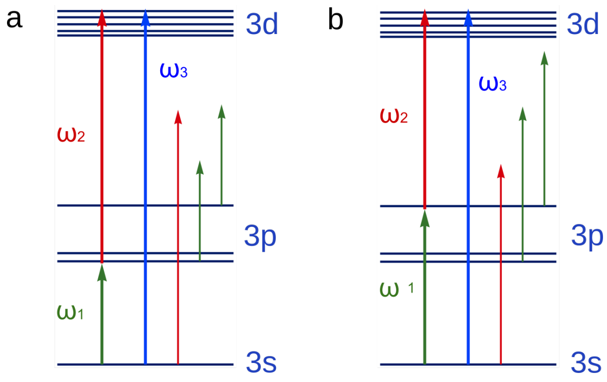

Figure S3 | Level schemes of methyloxirane with TRICC-field drivers. The TRICC-field wavelengths are (a) $\lambda_1 = 3438\,\mathrm{nm}$, $\lambda_2 = 1365\,\mathrm{nm}$, and $\lambda_3 = 977\,\mathrm{nm}$, or (b) $\lambda_1 = 2231\,\mathrm{nm}$, $\lambda_2 = 1726\,\mathrm{nm}$, and $\lambda_3 = 973\,\mathrm{nm}$ close to resonances for transitions between $3s$, (a) $3p_y$ and (b) $3p_x$, and $3d_{xy}$ states with energies from Table S2.

We benchmark the phase behaviour of the FID-active $3s$ state for a range of intensities up to those used in Figure S3. We compare the phase found analogously to Eq. (3) of the main text, but taking into account all of the states shown in Figure S3, as

$$\langle \delta E \rangle_O = \sum_{pd} \frac{\mathrm{Re}\{(\mathbf{d}_{sd}^* \cdot [\mathbf{d}_{sp} \times \mathbf{d}_{pd}])\,(\mathcal{E}_3^* \cdot [\mathcal{E}_1 \times \mathcal{E}_2]\,e^{i\phi})\}}{24\omega_{sp}\omega_{sd}} + \sum_p \frac{|\mathbf{d}_{sp}|^2 |\mathcal{E}_1|^2}{12\omega_{sp}} + \sum_d \frac{|\mathbf{d}_{sd}|^2 |\mathcal{E}_3|^2}{12\omega_{sd}}, \tag{S1}$$

and using the *ab initio* molecular dipoles from Table S3, with the numerical solution of the system of differential equations, further combined with orientation averaging. The orientation averaging for the numerical TDSE solution is done numerically, using Fibonacci numerical integration on a sphere [53] (with improved implementation as per Ref. [54]) for the orientation rotation axis, and cyclic rectangle-rule integration for the orientation rotation angle.

Figure S5 shows that there is a qualitative agreement between the numerical and analytical (S1) solutions averaged over molecular orientations for low intensities. For higher intensities the numerical phase starts oscillating around the analytical one, presenting partially resonant features. However, the values of the phase for both calculations are numbers of the same

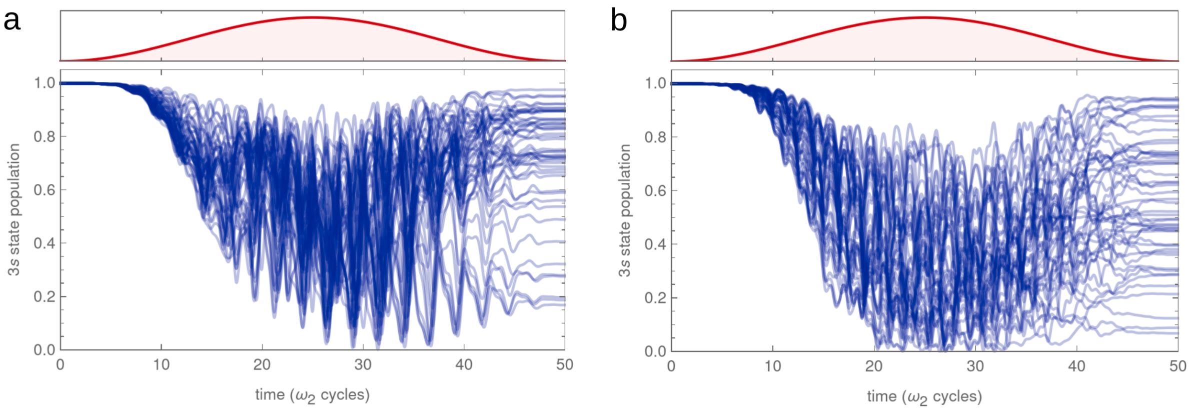

Figure S4 | FID-active state population dynamics. Population of the FID-active $3s$ state of methyloxirane for a random sample of orientations of the molecule during the TRICC pulse. The wavelengths of the TRICC-field components correspond to the schemes in Figure S3 (a) and (b), respectively. The intensities of the TRICC-field components are (a) $I_1 = 2\times10^{10}\ \mathrm{W/cm^2}$, $I_2 = 3.5\times10^{11}\ \mathrm{W/cm^2}$ and $I_3 = 2\times10^{11}\ \mathrm{W/cm^2}$, and (b) $I_1 = 1.5\times10^{10}\ \mathrm{W/cm^2}$, $I_2 = 1\times10^{11}\ \mathrm{W/cm^2}$ and $I_3 = 1.2\times10^{11}\ \mathrm{W/cm^2}$; the corresponding phases are $\phi_1 = \pi/3$, $\phi_2 = -\pi/3$, and $\phi_3 = \pi$. The pulse duration (intensity FWHM) is 25 cycles of the ω_2 field, and the focal waist of the UV beam is (a) $w_{UV} = 7\lambda_{UV}$ and (b) $w_{UV} = 6\lambda_{UV}$. The top panel shows the $\sin^2$ envelope of the TRICC field.

order and should result in the same strong effect. Moreover, to the extent that the calculations disagree, the analytical result used in the main text is an under-estimation of the numerical-TDSE result, which indicates that the results reported in the main text should, in real-world experiments, be achievable using lower driver intensities.

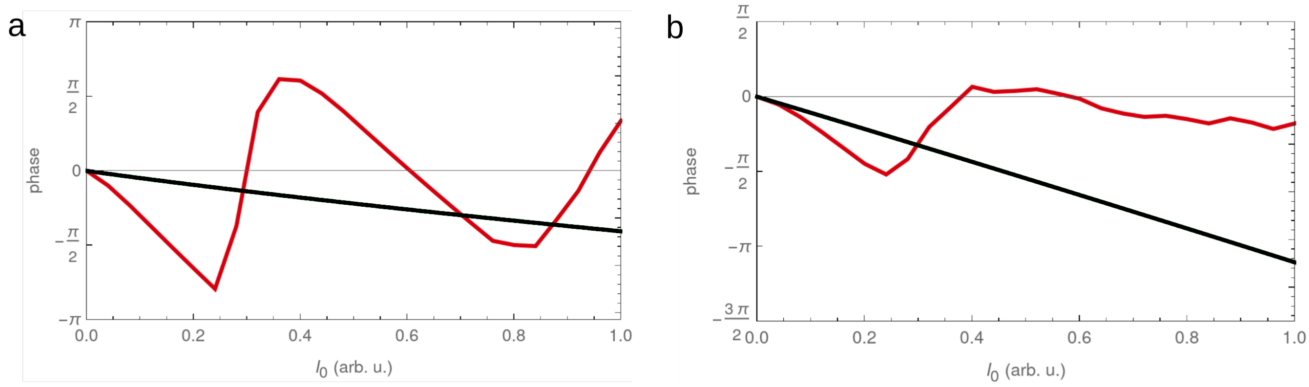

Figure S5 | Phase of FID-active state. The accumulated phase of the FID-active $3s$ state due to the TRICC dynamics, calculated fully analytically (S1) (black) and numerically (red), as a function of global intensity (i.e. each intensity used for Figure S4 is multiplied by I_0).

Additional results

Here we present the result for the enantiosensitive steering of FID by methyloxirane, which corresponds to the second scheme considered above (see Figure S3b), with the first scheme shown in the main text. We use TRICC-field parameters from Figure S4b. Figure S6a presents the phase of the FID-active $3s$ state accumulated during the TRICC pulse for different enantiomers of methyloxirane. The resulting deflection of the FID UV beam is presented in Figure S6b for both enantiomers. One can see that each enantiomer sends the beam at half a degree from the initial direction, which is easily observable experimentally.

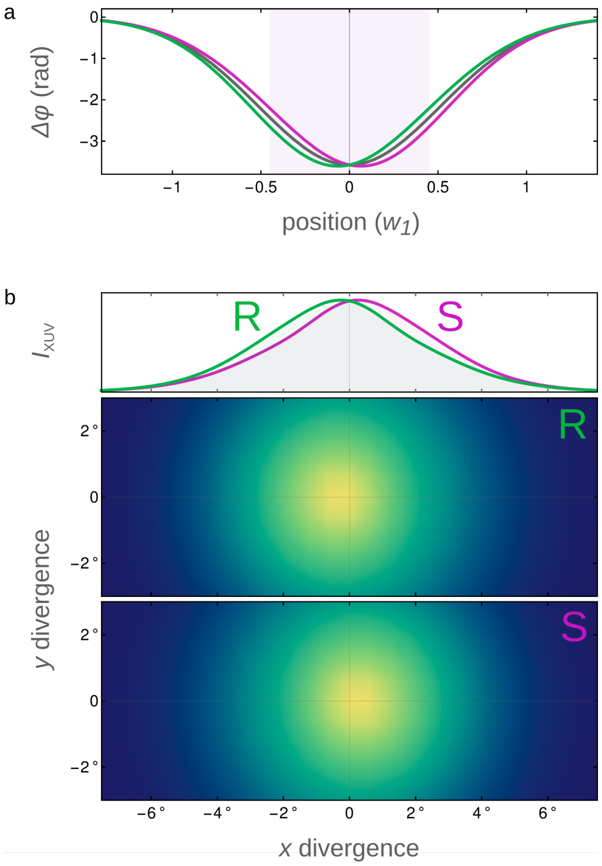

Figure S6 | Alternative FIDLE by methyloxirane. (a) Phase $\Delta\varphi$ accumulated in the FID-active Rydberg $3s$ state for R (green) and S (lilac) enantiomers; gray shows an achiral phase. (b) FID-beam divergence for each enantiomer, with a lineout of both on the top panel. The beams are deflected by about $0.5\,\mathrm{degrees}$. We use TRICC-field parameters corresponding to Figures S3b and S4b, with the TRICC-field components focused down to equal waists $w_1 = w_2 = w_3 = 1.2\lambda_1$.

Resonant case

Here we derive an analytical solution for the TDSE (see Methods) for the resonant case where there is zero detuning between the driving lasers and the corresponding molecular electronic transitions. We start from reducing the number of equations in the system (see Methods) by eliminating the complex amplitude of the state $|3\rangle$, which brings us to the system

$$\begin{aligned} i\ddot{c}_1 - \omega_{13}\dot{c}_1 + i|V_{13}|^2 c_1 &= -V_{12}e^{-i\omega_{12}t}\dot{c}_2 - i\left(V_{13}V_{23}^* + \omega_{23}V_{12}\right)e^{-i\omega_{12}t}c_2\,, \\ i\ddot{c}_2 - \omega_{13}\dot{c}_2 + i|V_{13}|^2 c_2 &= -V_{12}^* e^{i\omega_{12}t}\dot{c}_1 - i\left(V_{13}^*V_{23} + \omega_{13}V_{12}^*\right)e^{i\omega_{12}t}c_1\,. \end{aligned} \tag{S2}$$

For nonzero detunings, this system of differential equations does not have an analytical solution. However, for the resonant case, this system simplifies to

$$\begin{aligned} i\ddot{c}_1 + i|V_{13}|^2 c_1 &= -V_{12}\dot{c}_2 - iV_{13}V_{23}^* c_2\,, \\ i\ddot{c}_2 + i|V_{13}|^2 c_2 &= -V_{12}^*\dot{c}_1 - iV_{13}^*V_{23}c_1\,, \end{aligned} \tag{S3}$$

which can be solved within the ansatz $c_{1,2} = \tilde{c}_{1,2}e^{-\lambda t}$. The corresponding characteristic equation is

$$\lambda^4 + \lambda^2(|V_{12}|^2 + |V_{13}|^2 + |V_{23}|^2) - i\lambda(V_{12}V_{13}^*V_{23} + V_{12}^*V_{13}V_{23}^*) = 0\,,$$

with solutions

$$\lambda = \frac{2^{1/3}a}{\gamma}\begin{Bmatrix} e^{i\pi} \\ e^{i\pi/3} \\ e^{-i\pi/3} \\ 0 \end{Bmatrix} - \frac{\gamma}{2^{1/3}}\begin{Bmatrix} e^{-i\pi} \\ e^{-i\pi/3} \\ e^{i\pi/3} \\ 0 \end{Bmatrix}, \tag{S4}$$

where

$$
\begin{aligned}
a &= \frac{1}{3}(|V_{12}|^2 + |V_{13}|^2 + |V_{23}|^2)\,, \\
\gamma &= \left(ib + \sqrt{4a^3 - b^2}\right)^{1/3}\,, \\
b &= V_{12}V_{13}^*V_{23} + V_{12}^*V_{13}V_{23}^* = 2|V_{12}||V_{13}||V_{23}|\cos\phi\,, \\
\phi &= \phi_1 + \phi_2 - \phi_3\,.
\end{aligned}
\tag{S5}
$$

The trivial solution here addresses the stationary case, which is realised when $V_{12}V_{13}^*V_{23} = -V_{12}^*V_{13}V_{23}^*$ and corresponds to $\phi = \pi/2$.

If one of the interaction matrix elements (see Methods) is much smaller than other two, then the solutions λ can be written in the form:

$$
\lambda = 2i\left(\sqrt{a}\sin\phi_0 - \frac{b}{6a}\cos\phi_0\right)\,,
\tag{S6}
$$

where ϕ_0 is a constant phase for the different values of $\lambda_{1,2,3}$ in (S4). The complex amplitude of the state $|1\rangle$ in this case takes the form $c_1(t) = e^{i\delta Et}$, where

$$
\delta E = \frac{2|V_{12}||V_{13}||V_{23}|}{|V_{12}|^2 + |V_{13}|^2 + |V_{23}|^2}\cos\phi\cos\phi_0 - \frac{2}{\sqrt{3}}(|V_{12}|^2 + |V_{13}|^2 + |V_{23}|^2)^{1/2}\sin\phi_0\,.
\tag{S7}
$$

One can notice that the fact that λ in (S6) is imaginary leads to a pure energy shift of the excited state $|1\rangle$. Moreover, this energy shift (equivalently, phase shift) includes a linear dependence on the triple product of interaction matrix elements, which can be controlled through the relative phase ϕ between the TRICC-field components.

From the various solutions we found above in (S4) associated with different values of ϕ_0, we are interested in the solution corresponding to the situation where the initial population before the TRICC pulse is in the FID-active state, $|1\rangle$. In our case it is reasonable to assume that the longest wavelength field, $\mathcal{E}_2$, starts first, in which case the solution of interest corresponds to $\phi_0 = \pi$. In this case, the energy shift simplifies to

$$
\delta E = -\frac{2|V_{12}||V_{13}||V_{23}|}{|V_{12}|^2 + |V_{13}|^2 + |V_{23}|^2}\cos\phi\,,
\tag{S8}
$$

and, in the limit of $|V_{23}| \gg |V_{12}|, |V_{13}|$, to

$$
\delta E = -\frac{2|V_{12}||V_{13}|}{|V_{23}|}\cos\phi\,.
\tag{S9}
$$

However, in this case the orientation averaging can only be approached numerically, due to the presence of $|V_{23}|$ in the denominator. (Moreover, the approximation $|V_{23}| \gg |V_{12}|, |V_{13}|$ cannot hold uniformly for all molecular orientations, since $|V_{23}|$ depends on an inner product with $\boldsymbol{\mathcal{E}}_2$.) This raises the difficulty of analysis for this case as well as the numerical cost of computation (which nevertheless remains manageable).

REFERENCES AND NOTES

1. G. Palyi, *Biological Chirality* (Academic Press, 2019).

2. L. Pasteur, *Researches on the molecular asymmetry of natural organic products* (Alembic Club, 1905); ark:/13960/t77t0rb8m [translation from *Recherches sur la dissymétrie moléculaire des produits organiques naturels* (1861)].

3. Y. Tang, A. E. Cohen, Optical chirality and its interaction with matter. *Phys. Rev. Lett.* **104**, 163901 (2010).

4. D. Ayuso, O. Neufeld, A. F. Ordonez, P. Decleva, G. Lerner, O. Cohen, M. Ivanov, O. Smirnova, Synthetic chiral light for efficient control of chiral light–matter interaction. *Nat. Photonics* **13**, 866–871 (2019).

5. O. Neufeld, D. Ayuso, P. Decleva, M. Y. Ivanov, O. Smirnova, O. Cohen, Ultrasensitive chiral spectroscopy by dynamical symmetry breaking in high harmonic generation. *Phys. Rev. X* **9**, 031002 (2019).

6. D. Ayuso, A. F. Ordonez, M. Ivanov, O. Smirnova, Ultrafast optical rotation in chiral molecules with ultrashort and tightly focused beams. *Optica* **8**, 1243–1246 (2021).

7. A. F. Ordonez, O. Smirnova, Generalized perspective on chiral measurements without magnetic interactions. *Phys. Rev. A* **98**, 063428 (2018).

8. P. M. Rentzepis, J. A. Giordmaine, K. W. Wecht, Coherent optical mixing in optically active liquids. *Phys. Rev. Lett.* **16**, 792–794 (1966).

9. P. Fischer, D. S. Wiersma, R. Righini, B. Champagne, A. D. Buckingham, Three-wave mixing in chiral liquids. *Phys. Rev. Lett.* **85**, 4253–4256 (2000).

10. G. J. Simpson, Molecular origins of the remarkable chiral sensitivity of second-order nonlinear optics. *Chemphyschem* **5**, 1301–1310 (2004).

11. M. A. Belkin, Y. R. Shen, Non-linear optical spectroscopy as a novel probe for molecular chirality. *Int. Rev. Phys. Chem.* **24**, 257–299 (2005).

12. D. Patterson, M. Schnell, J. M. Doyle, Enantiomer-specific detection of chiral molecules via microwave spectroscopy. *Nature* **497**, 475–477 (2013).

13. Y.-Y. Chen, C. Ye, Q. Zhang, Y. Li, Enantio-discrimination via light deflection effect. *J. Chem. Phys.* **152**, 204305 (2020).

14. S. Eibenberger, J. Doyle, D. Patterson, Enantiomer-specific state transfer of chiral molecules. *Phys. Rev. Lett.* **118**, 123002 (2017).

15. C. Lux, M. Wollenhaupt, T. Bolze, Q. Liang, J. Köhler, C. Sarpe, T. Baumert, Circular dichroism in the photoelectron angular distributions of camphor and fenchone from multiphoton ionization with femtosecond laser pulses. *Angew. Chem. Int. Ed.* **51**, 5001–5005 (2012).

16. C. S. Lehmann, N. B. Ram, I. Powis, M. H. M. Janssen, Imaging photoelectron circular dichroism of chiral molecules by femtosecond multiphoton coincidence detection. *J. Chem. Phys.* **139**, 234307 (2013).

17. S. Beaulieu, A. Comby, D. Descamps, B. Fabre, G. A. Garcia, R. Géneaux, A. G. Harvey, F. Légaré, Z. Mašín, L. Nahon, A. F. Ordonez, S. Petit, B. Pons, Y. Mairesse, O. Smirnova, V. Blanchet, Photoexcitation circular dichroism in chiral molecules. *Nat. Phys.* **14**, 484–489 (2018).

18. A. F. Ordonez, O. Smirnova, Propensity rules in photoelectron circular dichroism in chiral molecules. II. General picture. *Phys. Rev. A* **99**, 043417 (2019).

19. R. Cireasa, A. E. Boguslavskiy, B. Pons, M. C. H. Wong, D. Descamps, S. Petit, H. Ruf, N. Thiré, A. Ferré, J. Suarez, J. Higuet, B. E. Schmidt, A. F. Alharbi, F. Légaré, V. Blanchet, B. Fabre, S. Patchkovskii, O. Smirnova, Y. Mairesse, V. R. Bhardwaj, Probing molecular chirality on a sub-femtosecond timescale. *Nat. Phys.* **11**, 654–658 (2015).

20. M. Pitzer, M. Kunitski, A. S. Johnson, T. Jahnke, H. Sann, F. Sturm, L. Ph. H. Schmidt, H. Schmidt-Böcking, R. Dörner, J. Stohner, J. Kiedrowski, M. Reggelin, S. Marquardt, A. Schießer, R. Berger,

M. S. Schöffler, Direct determination of absolute molecular stereochemistry in gas phase by Coulomb explosion imaging. *Science* **341**, 1096–1100 (2013).

21. C. Pérez, A. L. Steber, S. R. Domingos, A. Krin, D. Schmitz, M. Schnell, Coherent enantiomer-selective population enrichment using tailored microwave fields. *Angew. Chem. Int. Ed.* **56**, 12512–12517 (2017).

22. O. Neufeld, H. Hübener, A. Rubio, U. De Giovannini, Strong chiral dichroism and enantiopurification in above-threshold ionization with locally chiral light. *Phys. Rev. Res.* **3**, L032006 (2021).

23. F. Krausz, M. Ivanov, Attosecond physics. *Rev. Mod. Phys.* **81**, 163–234 (2009).

24. D. M. Villeneuve, Attosecond science. *Contemp. Phys.* **59**, 47–61 (2018).

25. J. Biegert, F. Calegari, N. Dudovich, F. Quéré, M. Vrakking, Attosecond technology(ies) and science. *J. Phys. B. At. Mol. Opt. Phys.* **54**, 070201 (2021).

26. S. Mukamel, *Principles of Nonlinear Optical Spectroscopy* (Oxford Univ. Press, 1995).

27. H. Rubinsztein-Dunlop, A. Forbes, M. V. Berry, M. R. Dennis, D. L. Andrews, M. Mansuripur, C. Denz, C. Alpmann, P. Banzer, T. Bauer, E. Karimi, L. Marrucci, M. Padgett, M. Ritsch-Marte, N. M. Litchinitser, N. P. Bigelow, C. Rosales-Guzmán, A. Belmonte, J. P. Torres, T. W. Neely, M. Baker, R. Gordon, A. B. Stilgoe, J. Romero, A. G. White, R. Fickler, A. E. Willner, G. Xie, B. McMorran, A. M. Weiner, Roadmap on structured light. *J. Opt.* **19**, 013001 (2016).

28. M. Chini, X. Wang, Y. Cheng, H. Wang, Y. Wu, E. Cunningham, P.-C. Li, J. Heslar, D. A. Telnov, S.-I. Chu, Z. Chang, Coherent phase-matched VUV generation by field-controlled bound states. *Nat. Photonics* **8**, 437–441 (2014).

29. S. Beaulieu, E. Bloch, L. Barreau, A. Comby, D. Descamps, R. Géneaux, F. Légaré, S. Petit, Y. Mairesse, Phase-resolved two-dimensional spectroscopy of electronic wave packets by laser-induced XUV free induction decay. *Phys. Rev. A* **95**, 041401 (2017).

30. H. Yun, J. H. Mun, S. I. Hwang, S. B. Park, I. A. Ivanov, C. H. Nam, K. T. Kim, Coherent extreme-ultraviolet emission generated through frustrated tunnelling ionization. *Nat. Photonics* **12**, 620–624 (2018).

31. S. Bengtsson, E. W. Larsen, D. Kroon, S. Camp, M. Miranda, C. L. Arnold, A. L'Huillier, K. J. Schafer, M. B. Gaarde, L. Rippe, J. Mauritsson, Space–time control of free induction decay in the extreme ultraviolet. *Nat. Photonics* **11**, 252–258 (2017).

32. L. Drescher, O. Kornilov, T. Witting, G. Reitsma, N. Monserud, A. Rouzée, J. Mikosch, M. J. J. Vrakking, B. Schütte, Extreme-ultraviolet refractive optics. *Nature* **564**, 91–94 (2018).

33. S. Bengtsson, J. Mauritsson, Ultrafast control and opto-optical modulation of extreme ultraviolet light. *J. Phys. B: At. Mol. Opt. Phys.* **52**, 063002 (2019).

34. L. Drescher, O. Kornilov, T. Witting, V. Shokeen, M. J. J.Vrakking, B. Schütte, Extreme-ultraviolet spectral compression by four-wave mixing. *Nat. Photonics* **15**, 263–266 (2021).

35. A. Olofsson, E. R. Simpson, N. Ibrakovic, S. Bengtsson, J. Mauritsson, Spatial control of extreme ultraviolet light with opto-optical phase modulation. *Opt. Lett.* **46**, 2356–2359 (2021).

36. S. Ghosh, G. Herink, A. Perri, F. Preda, C. Manzoni, D. Polli, G. Cerullo, Broadband optical activity spectroscopy with interferometric Fourier-transform balanced detection. *ACS Photonics* **8**, 2234–2242 (2021).

37. K. Y. Bliokh, F. Nori, Transverse and longitudinal angular momenta of light. *Phys. Rep.* **592**, 1–38 (2015).

38. K. Y. Bliokh, F. J. Rodríguez-Fortuño, F. Nori, A. V. Zayats, Spin-orbit interactions of light. *Nat. Photonics* **9**, 796–808 (2015).

39. L. D. Barron, L. Hecht, I. H. McColl, E. W. Blanch, Raman optical activity comes of age. *Mol. Phys.* **102**, 731–744 (2004).

40. A. Salam, W. J. Meath, On enantiomeric excesses obtained from racemic mixtures by using circularly polarized pulsed lasers of varying durations. *Chem. Phys.* **228**, 115–129 (1998).

41. V. Barone, M. Biczysko, J. Bloino, C. Puzzarini, Accurate molecular structures and infrared spectra of trans-2,3-dideuterooxirane, methyloxirane, and trans-2,3-dimethyloxirane. *J. Chem. Phys.* **141**, 034107 (2014).

42. D. L. Andrews, T. Thirunamachandran, On three-dimensional rotational averages. *J. Chem. Phys.* **67**, 5026–5033 (1977).

43. J. Ivanic, Direct configuration interaction and multiconfigurational self-consistent-field method for multiple active spaces with variable occupations. I. Method. *J. Chem. Phys.* **119**, 9364–9376 (2003).

44. J. Ivanic, Direct configuration interaction and multiconfigurational self-consistent-field method for multiple active spaces with variable occupations. II. Application to oxoMn(salen) and N_2O_4. *J. Chem. Phys.* **119**, 9377–9385 (2003).

45. M. W. Schmidt, K. K. Baldridge, J. A. Boatz, S. T. Elbert, M. S. Gordon, J. H. Jensen, S. Koseki, N. Matsunaga, K. A. Nguyen, S. Su, T. L. Windus, M. Dupuis, J. A. Montgomery, General atomic and molecular electronic structure system. *J. Comput. Chem.* **14**, 1347–1363 (1993).

46. C. Dykstra, *Theory and Applications of Computational Chemistry: The First Forty Years* (Elsevier, 2005).

47. T. H. Dunning, Gaussian basis sets for use in correlated molecular calculations. I. The atoms boron through neon and hydrogen. *J. Chem. Phys.* **90**, 1007–1023 (1989).

48. R. A. Kendall, T. H. Dunning Jr., R. J. Harrison, Electron affinities of the first-row atoms revisited. Systematic basis sets and wave functions. *J. Chem. Phys.* **96**, 6796–6806 (1992).

49. K. Kaufmann, W. Baumeister, M. Jungen, Universal Gaussian basis sets for an optimum representation of Rydberg and continuum wavefunctions. *J. Phys. B. At. Mol. Opt. Phys.* **22**, 2223–2240 (1989).

50. D. Sugic, M. R. Dennis, F. Nori, K. Y. Bliokh, Knotted polarizations and spin in three-dimensional polychromatic waves. *Phys. Rev. Res.* **2**, 042045 (2020).

51. C. Adams, *The Knot Book: An Elementary Introduction to the Mathematical Theory of Knots* (American Mathematical Society, 2004).

52. K. Kimura, S. Katsumata, Y. Achiba, T. Yamazaki, S. Iwata, *Handbook of HeI Photoelectron Spectra of Fundamental Organic Molecules. Ionization Energies, Ab Initio Assignments, and Valence Electronic Structure for 200 Molecules* (Halstead Press, 1981).

53. J. H. Hannay, J. F. Nye, Fibonacci numerical integration on a sphere. *J. Phys. A Math. Gen.* **37**, 11591–11601 (2004).

54. YARCHIK, https://mathematica.stackexchange.com/users/9469/yarchik, Package for fast spherical harmonic transform in Mathematica? [retrieved 17 September 2021]; https://mathematica.stackexchange.com/a/171856.
